# Ultra‐Wide Interlayered W_x_Mo_2x_S_y_ Alloy Electrode Patterning through High‐Precision Controllable Photonic‐Synthesis

**DOI:** 10.1002/advs.202403378

**Published:** 2024-07-29

**Authors:** Mengyao Tian, Xin Li, Aisheng Song, Chenyang Xu, Yongjiu Yuan, Qian Cheng, Pei Zuo, Sumei Wang, Misheng Liang, Ruoxi Wang, Tianbao Ma, Liangti Qu, Lan Jiang

**Affiliations:** ^1^ Laser Micro/Nano‐Fabrication Laboratory School of Mechanical Engineering Beijing Institute of Technology Beijing 100081 P. R. China; ^2^ Yangtze Delta Region Academy of Beijing Institute of Technology Jiaxing 314019 P.R. China; ^3^ Beijing Institute of Technology Chongqing Innovation Center Chongqing 401120 P. R. China; ^4^ State Key Laboratory of Tribology Tsinghua University Beijing 100084 P.R. China; ^5^ MOE Key Laboratory of Bioorganic Phosphorus Chemistry & Chemical Biology Department of Chemistry Tsinghua University Beijing 100084 P. R. China

**Keywords:** micro‐supercapacitors, photonic‐reduction, selective synthesis, temporally shaped femtosecond laser, W_x_Mo_2x_S_y_ alloys

## Abstract

Ultra‐thin 2D materials have great potential as electrodes for micro‐supercapacitors (MSCs) because of their facile ion transport channels. Here, a high‐precision controllable photonic‐synthesis strategy that provided 1 inch wafer‐scale ultra‐thin film arrays of alloyed W_x_Mo_2x_S_y_ with sulfur vacancies and expanded interlayer (13.2 Å, twice of 2H MoS_2_) is reported. This strategy regulates the nucleation and growth of transition metal dichalcogenides (TMDs) on the picosecond or even femtosecond scale, which induces Mo–W alloying, interlayer expansion, and sulfur loss. Therefore, the diffusion barrier of W_x_Mo_2x_S_y_ is reduced, with charge transfer and ion diffusion enhancing. The as‐prepared symmetric MSCs with the size of 100 × 100 µm^2^ achieve ultrahigh specific capacitance (242.57 mF cm^−2^ and 242567.83 F cm^−3^), and energy density (21.56 Wh cm^−3^ with power density of 485.13 W cm^3^). The established synthesis strategy fits numerous materials, which provides a universal method for the flexible synthesis of electrodes in microenergy devices.

## Graphical abstract

This work presents a novel photonic‐synthesis strategy for controllably patterned film electrodes in one step. It expands the time scale of chemical reaction modulation in the photosynthesis process and obtains an original alloyed electrode material with ultra‐wide interlayer spacing, which can improve the quality of microelectrodes and the flexibility of manufacturing technology.

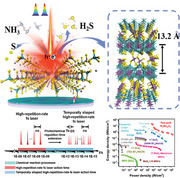

## 1 Introduction

With the development of the intelligent, electronic devices are gradually moving toward miniaturization,^[^ ^1^ ^]^ lightweight,^[^ ^2^ ^]^ portability,^[^ ^3^ ^]^ and multi‐functionality,^[^ ^4^ ^]^ increasingly powered by micro energy storage devices.^[^ ^5^ ^]^ Therefore, micro‐supercapacitors (MSCs) have attracted much attention due to their small size, high power density, fast charging/discharging process, and long cycle life.^[^ ^6^, ^7^ ^]^ The main limitation that restricts the development and application of MSCs is energy density.^[^ ^8^ ^]^ The way to further improve energy density while maintaining high rate performance and cycle life is the hotspot in MSCs research.^[^ ^9^, ^10^ ^]^

At present, the strategies for improving the energy density of MSCs mainly focus on material design and structural optimization.^[^ ^10^, ^11^ ^]^ For the perspective of material design, based on the principle of double‐layer energy storage formed by ion adsorption/desorption, electrode materials need to have a high specific surface area, high ion diffusion rate, and good conductivity.^[^ ^12^ ^]^ Ultra‐thin 2D materials are considered excellent energy storage materials due to their unique 2D layered structure, large specific surface area, and high planar electronic conductivity.^[^ ^13^, ^14^ ^]^ Expanding the interlayer spacing promotes the storage and transport of ions and electrons in 2D materials, which could suppress material volume expansion/contraction, and reduce adsorption/intercalation barriers.^[^ ^15^, ^16^ ^]^ The existing methods for expanding the interlayer spacing of 2D materials include doping with heteroatoms (such as N, O, etc.), intercalation with alkali metal ions (such as lithium ions, sodium ions), small molecule intercalation, polymer intercalation.^[^ ^17^, ^18^, ^19^, ^20^ ^]^ These methods could control the band structure, electronic structure, and crystal structure of materials through electron/hole injection, optimizing the electrochemical performance of 2D materials.^[^ ^21^ ^]^ For the perspective of structural optimization, reducing feature size^[^ ^22^ ^]^ is a major strategy to improve the specific capacity of MSCs, which can effectively regulate the reaction kinetics of energy storage devices, and improve the area‐specific capacitance and energy density of devices.^[^ ^23^ ^]^ Therefore, the miniaturization and high‐precision processing of MSCs is crucial. Reports have shown that methods such as laser direct writing,^[^ ^24^ ^]^ photolithography,^[^ ^25^ ^]^ and focused ion beam etching^[^ ^26^ ^]^ can achieve micro and submicron‐level patterned processing of electrode materials. However, at present, it is difficult to achieve collaborative control between material synthesis and structural miniaturization. The expansion of interlayer spacing and high‐precision patterned synthesis of ultra‐thin 2D materials are difficult to complete in one step.^[^ ^27^ ^]^ Therefore, there is an urgent need for a micro patterned synthesis technology that can balance material synthesis, regulation, and electrode patterning processing, to achieve controllable preparation of high‐performance ultra‐thin 2D materials and one‐step patterned manufacturing of high‐energy density MSCs. Laser processing could flexibly perform additive/subtractive/modified manufacturing^[^ ^28^ ^]^ and modulate material physical and chemical properties^[^ ^29^ ^]^ due to its simplicity of processing, material adaptability, and wide tunability, which covers millimeter to nanometer.^[^ ^30^ ^]^ Many excellent studies^[^ ^31^ ^]^ have demonstrated that laser fabrication can play an important role for the processing synthesis and property modulation of 2D materials such as graphene or graphene‐like materials.^[^ ^32^ ^]^

Here, we propose a high‐precision light‐controlled atomic level material patterned growth strategy, achieving a one‐step patterned synthesis of 2D ultrathin films. Alloyed W_x_Mo_2x_S_y_ thin films with ultra‐wide interlayer spacing were obtained successfully, which were used to prepare thin supercapacitors with ultra‐high specific capacitance and energy density. We have built a high repetition rate temporally shaped photosynthesis system and achieved a direct patterned synthesis of high crystallinity alloyed W_x_Mo_2x_S_y_ thin films from two to a few layers. We intervene in the chemical reaction process by regulating the photochemical synthesis microcavities of instantaneous high temperature and high pressure, injecting more electrons/holes in the early stage of material nucleation and growth, inducing interlayer expansion and sulfur vacancy formation, and selectively and effectively converting the target precursor film from (NH_4_) _2_MoS_4_ and (NH_4_) _2_WS_4_ to an alloyed W_x_Mo_2x_S_y_ film. Local processing based on the real‐time laser can generate various patterned W_x_Mo_2x_S_y_ structures of different scales. Ultra‐thin films can form patterns on 1‐inch wafers under environmental conditions without damaging different types of substrates, including SiO_2_/Si, Au, and PET. Multiple materials such as MoS_2_, WS_2_, and W_x_Mo_2x_S_y_ have been synthesized successfully. The manufacturing of multi‐shaped MSCs such as cross fingers, concentric circles, and parallel bars have been achieved. Among the prepared MSCs, the four‐finger forked W_x_Mo_2x_S_y_ thin film exhibited the best electrochemical performance due to its ultra‐wide interlayer spacing of 13.2 Å and alloy lattice structure. Benefiting from enhanced volume transfer and electron transfer, the 10 nm W_x_Mo_2x_S_y_ film produced provides an excellent specific capacity of 242.57 mF cm^−2^ and 242 567.83 F cm^−3^, as well as ultra‐high energy density of 21.56 Wh cm^−3^ at a scanning rate of 5 mV s^−1^. The rapid diffusion kinetics and transport behavior of H^+^ in the W_x_Mo_2x_S_y_ electrode were confirmed by electrochemical measurements and density functional theory (DFT) calculations. The expanded and ultra‐wide interlayer spacing of W_x_Mo_2x_S_y_ reduced ion diffusion resistance and increased the available and accessible active surface area. The alloy composition could lower the electron conduction barrier.

## 2 Results and Discussion

### 2.1 Femtosecond Laser Temporally Shaped Photonic‐Reduction

**Figure 1:**
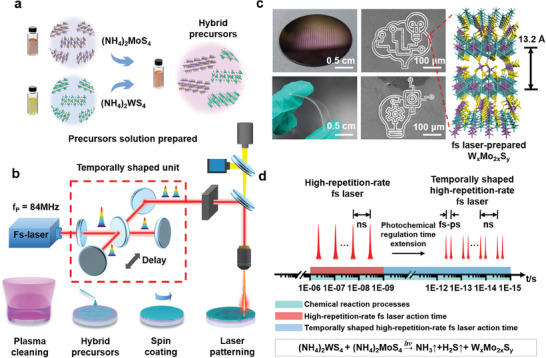
Schematic diagram of temporally shaped high‐repetition‐rate femtosecond laser one‐step synthesis and pattern reduction processing. a) Schematic diagram of the hybrid precursor (NH_4_)_2_MoS_4_ and (NH_4_)_2_WS_4_ solution. b) Schematic diagram of the film prepared and the temporally shaped high‐repetition‐rate femtosecond laser processing. c) The images of patterned films in different scales. 1) Photograph of a W_x_Mo_2x_S_y_ film array on a 1 in. SiO_2_/Si wafer and flexible PET substrate. 2) Scanning electron micrographs of patterned W_x_Mo_2x_S_y_ films. 3) Schematic diagram of as‐prepared W_x_Mo_2x_S_y_ films with 13.2 Å interlayer space. d) Schematic diagram of the regulatory mechanism by temporally shaped photo‐reduction.

**Figure** **1** demonstrates the strategy of selective photo‐reduction of alloyed W_x_Mo_2x_S_y_ with the help of a temporally shaped femtosecond laser. As shown in Figure 1a, the synthesis of patterned MoS_2_, WS_2_, and alloyed W_x_Mo_2x_S_y_ thin films started from the complete and uniform coverage of each other's precursor films. First, we mixed the ammonium tetrathiomolybdate and ammonium tetrathiotungstate as precursors for MoS_2_ and WS_2_, and then prepared hybrid ammonium tetrathiomolybdate/ammonium tetrathiotungstate thin films by spin–coating. Then we constructed a temporally shaped high repetition frequency ultrafast laser photochemical patterning synthesis system as shown in Figure 1b, with the repetition frequency of the output laser at 84 MHz. The Michelson interferometer device was proposed to cleave the laser pulse and regulate the optical delay and the merging of the laser pulses to achieve the high‐precision controllable photonic‐synthesis of the materials with patterning meanwhile. Benefiting from the instantaneous high temperature and high pressure,^[^ ^33^ ^]^ non‐contact,^[^ ^34^ ^]^ tight focusing,^[^ ^35^ ^]^ and good controllability of nucleation sites of the temporally shaped photochemical patterning synthesis system we constructed, the material applicability of this method is relatively broad, and it can be used to process almost any precursor that can be thin‐film. It could form an arbitrary pattern on a rigid or flexible substrate as shown in Figure 1c, which can be used to generate high‐precision, multi‐patterned, and flexible synthesis of thin films by controlling the focusing laser parameters, achieving flexible and controllable synthesis. A Michelson interferometer is introduced into the processing optical path (Figure 1b), and a thin‐film beam splitter is used to split the incident laser pulse into reflected and transmitted light with an energy ratio of ≈1:1, which is then reflected by the two mirrors. Then the two pulses returned to the original path through the thin‐film beam splitter and re‐combined into the processing optical path. By adjusting the position of the two mirrors, the optical range between the reflected light and the transmitted light was controlled. The two sub‐pulses can form a temporally shaped femtosecond laser pulse train adjustable in the range of femtoseconds to picoseconds. As shown in Figure 1d, the coupling of the temporally shaped module and the high repetition rate laser could shorten the pulse interval from 11.9 ns (1/84 MHz) to fs, which is much smaller than the physical and chemical characteristic time of most materials. It means the extension of the photochemical reaction modulation range from nanoseconds to picoseconds and even femtoseconds. It enables the synthesis system to obtain alloyed W_x_Mo_2x_S_y_ thin films with high surface quality, modulated in terms of elemental ratios, lattice states, and other physicochemical properties of the materials at the same time. It is noteworthy that the alloyed W_x_Mo_2x_S_y_ material synthesized by this strategy has an extraordinarily large layer spacing (13.2 Å) at a sub‐pulse delay of 1 ps after laser cleavage.

Using this ultrafast and controllable patterned thin film synthesis system, we synthesized single materials MoS_2_ and WS_2_. Meanwhile, composite alloyed materials W_x_Mo_2x_S_y_ were also synthesized. At the beginning of the experiments, we compared the effects of different laser repetition frequencies and different laser pulse widths on the synthesis of a single thin film of MoS_2_ as shown in Figure S1 (Supporting Information). We found that the laser repetition frequency had a significant effect on the crystallization of the MoS_2_ material. It is observed that the laser repetition frequency has an obvious effect on the crystallization of MoS_2_ material. The MoS_2_ material synthesized under the femtosecond laser excitation with a repetition frequency of 84 MHz possesses a more obvious $E_{2g}^{1}$ peaks of the in‐plane vibration and A_1g_ peaks of the interlayer vibration, which is attributed to the fact that the pulsed laser irradiation among the primitive tetrathiomolybdenum ammonium‐molybdate precursor film constitutes a transient high‐temperature, high‐pressure synthesizing environment with a diameter of the sub‐micrometer to the micrometer level. The laser repetition frequency decides the pulsed laser single pulse energy. Compared to the 1 kHz laser, the 84 MHz laser has a lower single pulse, a more uniform energy deposition, and a better continuity, which was more conducive to the crystallization of thin film materials and continuous growth. The single pulse energy of the 1 kHz femtosecond laser is relatively large after focusing and is likely to change the material into an amorphous state or remove it, destroying the continuity of the thin film. The energy of the 1 kHz picosecond laser is absorbed strongly, which produces a large amount of debris that is not favorable to the surface quality of the film. In summary, a laser with high repetition frequency and low single pulse energy is more favorable for the crystallization of the material to obtain continuous and smooth films.

The change of the local transient high temperature and high‐pressure environment of the synthesis system can be realized by controlling the laser parameters. We explored the effect of the laser fluence on the crystalline phases and elemental compositions of the synthesized materials, as shown in Figure S2 (Supporting Information). The in‐plane $E_{2g}^{1}$ peaks (384 cm^−1^) and the out‐of‐plane A_1g_ peaks (404 cm^−1^) are observed for laser fluence in the range of 0.012 J cm^−2^–0.024 J cm^−2^, which correspond to the characteristic peaks of crystalline 2H MoS_2_.^[^ ^36^ ^]^ The additional peaks located at 200, 225, and 355 cm^−1^ (marked by the red box), which correspond to the characteristic Raman peaks of the 1T' ‐phase MoS_2_,^[^ ^37^ ^]^ are observed at a laser fluence of 0.018 J cm^−2^. The films synthesized at 0.018 J cm^−2^ and two nearby fluences were selected for X‐ray photoelectron spectroscopy measurements. The results of the measurements (red dotted curves) can be deconvoluted into the characteristic Mo^5+^ peaks located at 230.3 eV (Mo^5+^ 3d_5/2_) and 233.5 eV (Mo^5+^ 3d_5/2_), the characteristic peaks located at 229.2 eV (Mo^4+^ 3d_5/2_) and 232.3 eV (Mo^4+^ 3d_3/2_), Mo^4+^ characteristic peaks at 229.2 eV (Mo^4+^ 3d_5/2_) and 232.3 eV (Mo^4+^ 3d_3/2_) and Mo^3+^ characteristic peaks at 228.3 eV (Mo^3+^ 3d_5/2_) and 231.4 eV (Mo^3+^ 3d_3/2_).^[^ ^37^, ^38^ ^]^ The three elemental ratios of Mo, S, and O were extracted from them with laser fluence. The Mo/S elemental ratios showed a tendency to increase and then decrease with the increase of laser fluence and reached the maximum Mo/S = 0.63 at 0.018 J cm^−2^. We speculate that as the laser fluence of the synthesis system increases, the bonding of Mo and S elements gradually increases. After exceeding a laser fluence of 0.018J  cm^−2^, the laser ablates and breaks the bonding of the synthesized surface MoS_2_, causing an increase in S vacancies and a decrease in the Mo/S atomic ratio.^[^ ^39^ ^]^ The S element vacancies gradually increased, resulting in a decrease in the Mo/S atomic ratio. Based on the variation of the area ratio of the characteristic peaks of XPS deconvolution with the laser fluence, we qualitatively compared the change of the proportion of Mo^3+^ characteristic peaks located at 228.3 eV (Mo^3+^ 3d_5/2_) and 231.4 eV (Mo^3+^ 3d_3/2_). The proportion of Mo^3+^ showed an upward trend in the stage of 0.012–0.018 J cm^−2^. The proportion of Mo^3+^ showed an increasing trend in the stage of 0.018 J cm^−2^ and decreased at 0.018–0.024 J cm^−2^. This is due to the fact that after the laser fluence is greater than 0.018 J cm^−2^, the thermal effect brought by the laser with larger energy will modify the surface of the 1T' phase MoS_2_ into the conventional phase MoS_2_ of 2H, which will be changed into the conventional phase of 2H.^[^ ^40^ ^]^ Continued increase in laser fluence removes the material as shown in Figure S3 (Supporting Information). The synthesized material undergoes this modification–ablation process layer by layer, and thus the percentage of 1T' phase decreases at higher laser fluence.

To further validate the multiphase synthesis capability of our synthesis system, high‐resolution transmission electron microscopy (HRTEM) was further used to characterize the atomic structure and to resolve the crystalline phase of L‐MoS_2_ (laser fluence of 0.018 J cm^−2^). The EDS elemental distribution and spectra shown in Figure S4 (Supporting Information) verified that our synthesized thin film material is lamellar and mainly composed of Mo and S. Further a triangular lattice was observed through the high‐resolution images shown in Figure S5 (Supporting Information). The overall lattice structure indicates the proper laser parameters for the synthesis brought no obvious defects and deformations. The 2H‐phase MoS_2_ obtained by our synthesis method agrees with previous studies, possessing a layer spacing of 6.2 Å.^[^ ^41^, ^42^ ^]^ The 1T'‐phase MoS_2_ possesses an enlarged layer spacing of 8.5 Å.^[^ ^37^ ^]^ The results are consistent with those of previous studies. The dislocations of the two S atoms can be observed in the brightness distribution generated from the fast Fourier inversion transform (FFT), demonstrating the semiconductor 2H phase and the metallic 1T' phase. All the above experimental results indicate that the lattice structure of the prepared MoS_2_ films is heterogeneous, consisting of 1T' and 2H phases, and that our synthesis system possesses homogeneous multiphase synthesis capability (Figures S6 and S7, Supporting Information).

The high repetition rate temporally shaped photosynthesis system was used to expose the precursor in micro‐regions, with a high‐temperature and high‐pressure synthesis environment. The modulation of different laser parameters can affect the nucleation and crystallization of the material, as shown in Figure S8 (Supporting Information). In the initial exploratory experiments, we found that with the increase of the laser power, the intensity of the in‐plane $E_{2g}^{1}$ peak (384 cm^−1^) and out‐of‐plane A_1g_ peak (404 cm^−1^) of MoS_2_ increases first and then decreases with increasing laser power, reaching a maximum density at 10 µW. The micro‐synthesis cavity with different moving speeds caused by the laser spots in different scanning speeds had a large effect on the crystallinity, which appeared the best state at 50 µm s^−1^ among the four moving speeds. Whereas the main effect of the introduced temporally shaped module on the material is reflected in the modulation of the lattice structure. The introduction of temporally shaped does not disrupt the crystallinity when compared to the single‐pulse (no temporally shaped module) synthesized material, as shown in Figure S9 (Supporting Information).

### 2.2 Multiscale Patterned Film Within One‐Step Synthesis

**Figure 2:**
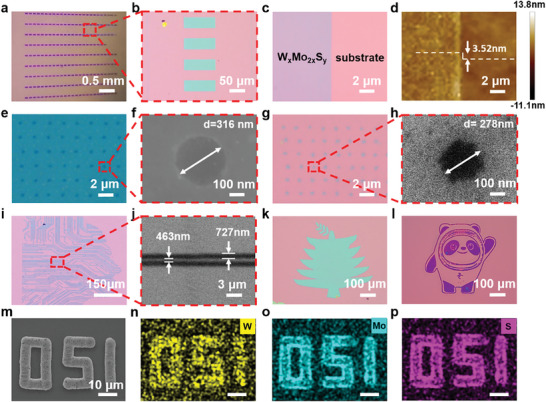
Structural characterization of the W_x_Mo_2x_S_y_ nanocomposites. a) Photographs of W_x_Mo_2x_S_y_ arrays on SiO_2_/Si substrate. b) Optical Microscope image of few‐layer W_x_Mo_2x_S_y_ films. c,d) Optical Microscope and atomic force micrographs of few‐layer W_x_Mo_2x_S_y_ film with the height of 3.52 nm. e,f) Punch on W_x_Mo_2x_S_y_ film with 316 nm beyond the diffraction limit in the positive phase. g,h) Synthesis of W_x_Mo_2x_S_y_ nanorods with 278 nm beyond the diffraction limit in the negative phase. i,j) Synthesis of W_x_Mo_2x_S_y_ pattern with 463 nm resolution and 727 nm linewidth. k,l) Graphic synthesis of W_x_Mo_2x_S_y_ film with the shape of a tree and panda. m–p) EDS mapping images of the synthesized W_x_Mo_2x_S_y_ pattern(delay‐time = 1 ps).

Compared with other methods of synthesizing 2D thin film materials, the photosynthesis system can construct a synthesized microcavity by instantaneous high temperature and high pressure in the micrometer scale region at room temperature, which does not require a harsh high‐vacuum environment. Moreover, the size and path of the synthesized microcavity transient can be flexibly regulated by adjusting the pulse train parameter, motion parameter, and objective focusing parameter. This ability to synthesize one‐step patterned films at room temperature is of great interest in the field of device fabrication. As shown in **Figure** **2**a,b, we performed the synthesis of 50 µm × 100 µm W_x_Mo_2x_S_y_ composite thin film arrays on 1‐inch SiO_2_/Si wafers. After dissolving away the precursor films with solvent (Dimethylformamide, DMF), it can be seen that the thin film arrays are aligned neatly with clear edges. It is observed by optical microscopy and atomic force microscopy that the film edges are straight and well‐resolved from the substrate with good surface quality. The synthesized thickness is down to 3.52 nm (more thickness in Figure S10, Supporting Information) based on ensuring the film continuity as shown in Figure 2c,d. Characterized by the subsequent high‐resolution transmission electron microscopy, the W_x_Mo_2x_S_y_ layer has a space of 13.2 Å, indicating the synthesis of an approximate two‐layer W_x_Mo_2x_S_y_. The main factors affecting the thickness of film synthesis in the photochemical synthesis system are precursor film thickness and laser fluence. We also explored the effect of precursor thickness and laser fluence on film thickness (single irradiation molding) as shown in Figure S11 (Supporting Information), which approximates a linear correlation. Due to the very small single‐pulse energy of the laser, the 3D dimensions of the instantaneous synthesized microcavity were relatively miniature, which means it is difficult to transfer the energy completely to the interface between the thick precursor membrane (more than 50 µm) and the substrate. Therefore, the synthesis of thicker membranes requires a second irradiation of the laser or a synthesis microcavity with a larger single pulse energy.

Figure 2e–j demonstrates the processing capability of this laser synthesis system for 0D and 1D feature patterns in addition to 2D films. We tight‐focused with a 50x objective lens (NA = 0.8) on the precursor film with a thickness of 4 µm, and obtained the 0D nanodot arrays with a minimum diameter of 316 nm. After the film was cleaned by DMF to dissolve the precursor, we obtained 0D nanodot W_x_Mo_2x_S_y_ film arrays in the 0D super diffraction limit with a minimum diameter of 278 nm, which is the smallest synthesis unit of our current photosynthesis system. Then we synthesized the human brain‐like line pattern shown in Figure 2i by laser direct‐write patterned synthesis. The average line width is 727 nm and the spacing between lines is about 463 nm from the pictures taken by scanning electron microscope. The resolution of the 0D thin‐film feature pattern of the photosynthesis system can be reduced to 278 nm. The resolution of the 1D thin‐film feature pattern can be reduced to 463 nm. With such points and lines as synthesis units, we synthesized different flexible and variable patterns such as the tree shown in Figure 2k, the panda pattern shown in Figure 2l, and the numbers shown in Figure 2m. The scanned images of the EDS element distribution indicate that the elements of W, Mo, and S are uniformly distributed on the numbers (Figure S12, Supporting Information), which proves that the high repetition rate temporally shaped photosynthesis system successfully realizes the one‐step patterning thin film W_x_Mo_2x_S_y_ synthesis.

### 2.3 Characterization and Analysis of Laser‐Induced W_x_Mo_2x_S_y_ Alloys Materials

**Figure 3:**
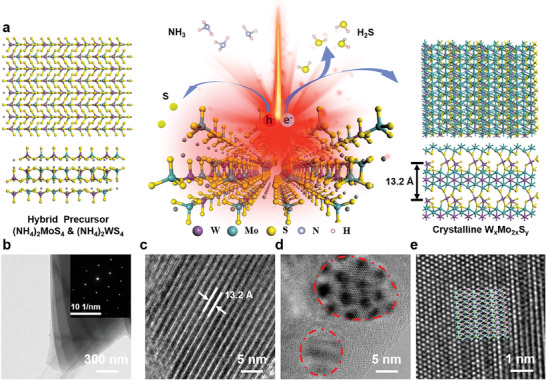
Schematic diagram of the lattice transition process of the one‐step synthesis. a) Schematic diagram of lattice conversion from the hybrid precursor (NH_4_)_2_MoS_4_ and (NH_4_)_2_WS_4_ to W_x_Mo_2x_S_y_ with 13.2 Å interlayer space. b) High‐resolution transmission electron micrographs (HRTEM) of the laser‐synthesized layered material. c) High‐resolution transmission electron micrographs (HRTEM) of interlayer expanded films. d) High‐resolution transmission electron micrographs (HRTEM) of composite films. e) High‐resolution transmission electron micrographs (HRTEM) of composite films with lattice structure.

**Figure** **3**a demonstrates the chemical reaction process and the transformation of the material lattice structure of the mixed ammonium tetrathiomolybdate and ammonium tetrathiotungstate precursor membranes inside the high repetition rate temporally shaped photosynthesis microcavity with the local transient high‐temperature and high‐pressure environment. The following chemical reaction changes occurred within the photosynthesized microcavity:

(1) $$\begin{array}{ccc} {\left(NH_{4}\right)}_{2}WS_{4}\; & + & \;{\left(NH_{4}\right)}_{2}\mathrm{Mo}S_{4}\;\overset{h\nu }{\to }\;NH_{3}↑+H_{2}S↑\\ & & \;+\;W_{x}Mo_{2x}S_{y}\;+S↓ \end{array}$$

Under the excitation of the high repetition rate laser, electron‐hole pairs appear in the thin film material from the pristine state of a mixture on the left side of Figure 3a. The S^2−^ loses its electrons and rises in valence to S^0^ and H_2_S, which is verified by the S^0^ peaks in X‐ray photoelectron spectroscopy.^[^ ^43^ ^]^ The Mo^6+^ and W^6+^ get electrons to drop in valence and alloy due to a high degree of overlap in the nucleation sites, forming the novel material W_x_Mo_2x_S_y_. The pulse spacing (10^−9^ s) of the high repetition rate femtosecond laser (84 MHz) is in the range of the photochemical reaction time (10^−9^–10^−6^ s) of the precursor material and thus induces nucleation and crystallization growth. However, its regulation of the chemical reaction pathway is also on the nanosecond scale. The subsequent pulses continue to act for nanoseconds after the first laser pulse excites a large number of electron‐hole pairs, which is far beyond the time scale of electron and lattice heating (10^−15^–10^−12^ s). We incorporate a temporally shaped module (10^−15^−10^−12^ s) to shorten the intervention time of photochemistry to the order of femtoseconds and picoseconds for controlled nucleation, crystallization, and growth. After the first sub‐pulse excites the electron hole, the second sub‐pulse arrives before the material undergoes a reaction/phase transition (10^−15^–10^−12^ s), exciting more electron holes to participate in the transformation of the chemical reaction path. Thus, the method realizes the intervention in the chemical reaction path through electronic excitation and modulation on the time scale, which leads to different previous products. Based on the elemental ratios in the X‐ray photoelectron spectra we summarize the atomic ratios of W, Mo, and S synthesized at different laser energies (Table S1, Supporting Information), and find that approximately W: Mo is always equal to a 1:2. The amount of S decreases with the increase of the laser energy, presumably because a larger laser fluence would have an ablation removal of synthesized materials, which induces more S vacancies, which is reflected in previous studies.^[^ ^39^ ^]^ So we summarize the alloyed material in the right panel of Figure 3a as W_x_Mo_2x_S_y_, with x and y corresponding to the elemental ratios in Table S1 (Supporting Information). Surprisingly, the synthesized material is well crystallized, presenting a very good layer structure as shown in Figure 2b, and a significantly enlarged layer spacing, where the maximum layer spacing is 13.2 Å at *x* = 1, *y* = 1.5 (Figure 3c), as shown in Figure 3d,e. Among the high‐resolution transmission electron microscopy, we found traces of the alloyed composite of the thin film material. We observed the appearance of the dislocation‐like lattice structure with a regular arrangement of W and Mo atoms in the high‐resolution lattice structure, demonstrating the synthesis of W_x_Mo_2x_S_y_ materials. Combining the lattice structure from high‐resolution transmission electron microscopy and the atomic ratios from XPS characterization, we have used first‐principles calculations to relax the crystalline WMo_2_S_1.5_ lattice structure with an approximate layer spacing of 13.2 Å. The lattice structure is shown in the lower right corner of Figure 3a.

**Figure 4:**
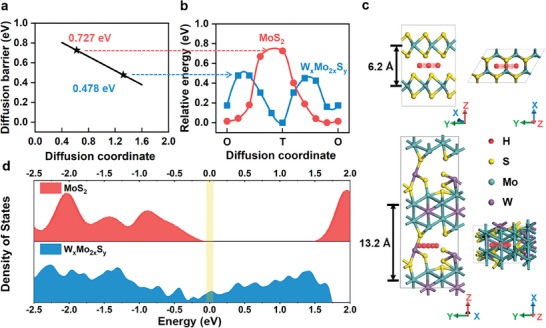
Theoretical simulation of H^+^ diffusion in W_x_Mo_2x_S_y_ and pristine MoS_2_ with different interlayer distances. a) The energy barrier for H^+^ diffusion continuously decreases as the interlayer distance increases. b) Potential energy diagram for H^+^ migration at interlayer space of c) *d* = 6.2 Å for MoS_2_, and c) *d* = 13.2 Å for W_x_Mo_2x_S_y_. d) The density of states of pristine MoS_2_ and W_x_Mo_2x_S_y_.

To verify that our prepared WMo_2_S_1.5_ layer spacing spreading is favorable for cation migration, we calculated the diffusion behaviors of H in WMo_2_S_1.5_ and pristine 2H MoS_2_ using the density‐functional theory (DFT) method, where the layer spacing is 13.2 Å for WMo_2_S_1.5_ and 6.2 Å for 2H MoS_2_. Observing the ion diffusion paths, the octahedral sites (O sites) are more favorable than tetrahedral sites (T sites) for H embedding and adsorption from an energetic point of view. This is consistent with previous observations on bulk MoS_2_.^[^ ^44^, ^45^ ^]^ As shown in **Figure** **4**a, the diffusion energy barriers are 0.727 eV for 2H MoS_2_ and 0.478 eV for WMo_2_S_1.5_, corresponding to the highest migration potential in Figure. 4b. Under the assumption of the standard Arrhenius expression (D∝e^−Ea/RT^), this reduction in barrier corresponds to an acceleration of H diffusion by a factor of 10^14^. Figure 4b visually shows the potential energy diagram for H migration. At the 2H MoS_2_ material with a layer spacing of *d* = 0.62 nm, the total energy increases dramatically as H diffuses from the O site to the T site. On the contrary, at the large spacing of *d* = 1.32 nm for the WMo_2_S_1.5_, only a small amount of energy is required for H migration away from the O site. With the increase of layer spacing, the adsorption between H and MoS_2_ is gradually weakened, and the larger spacing of *d* = 1.32 nm makes H no longer stay in the middle of the two layers, which is more similar to the adsorption of H on monolayer MoS_2_.^[^ ^46^ ^]^ The larger layer spacing and S vacancies effectively reduce the spatial resistance of H, lowering the energy barrier. And the rest of the diffusion path does not add additional energy barriers. The 2H MoS_2_ has an obvious band gap at the same position, while the predicted density of states (DOS) of WMo_2_S_1.5_ exhibits a larger state at the Fermi energy level (Figure 4d), suggesting that the higher electronic conductivity of WMo_2_S_1.5_ is a consequence of alloying as well as having the metallic phase 1T. From the above simulation results, it can be summarized that the interlayer expansion of WMo_2_S_1.5_ prepared by the photosynthesis system is a powerful technique to improve the cation diffusion kinetics in the lamellar body of the material. The low H migration barrier and high electronic conductivity ensure fast charge storage kinetics and excellent rate capability.

**Figure 5:**
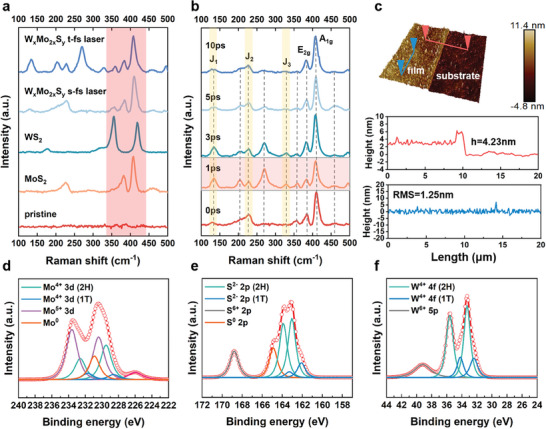
Structural characterization of the W_x_Mo_2x_S_y_ nanocomposites. a) Raman spectra contrast the highlighting of the shift of the alloy W_x_Mo_2x_S_y_ mode in different laser‐synthesis materials. b) Raman spectra contrast of the alloy W_x_Mo_2x_S_y_ mode among different delays of t‐fs laser‐synthesis processing. c) Atomic force micrographs of 3D surface morphology of W_x_Mo_2x_S_y_. d–f) X‐ray photoelectron spectra results of Mo 3d, S 2p, and W 4f orbitals of the synthesized W_x_Mo_2x_S_y_ (delay‐time = 1 ps).

To further reveal the detailed crystal structure features of the synthesized films, we have used Raman spectroscopy (Raman), X‐ray photoelectron spectroscopy (XPS) to characterize the single materials MoS_2_, WS_2_, and the alloyed composite material W_x_Mo_2x_S_y_ prepared under this synthesis system. As shown in **Figure** **5**a, The vertical red area covers the range of the main characteristic peaks of the three materials, which are the *A*_1*g*_ and $E_{2g}^{1}$ modes.Compared to MoS_2_, the *A*_1*g*_ mode of W_x_Mo_2x_S_y_ (≈410 cm^−1^) is shifted by ≈2 cm^−1^ in the high‐energy direction, and the $E_{2g}^{1}$ mode of MoS_2_‐like (≈381 cm^−1^) is shifted by ≈1 cm^−1^ in the low‐energy direction, resulting in a stronger separation of the planar vibrational characteristic peaks (≈29 cm^−1^) signal modes than that of MoS_2_ (≈26 cm^−1^). Compared with WS_2_, the characteristic peak of the *A*_1*g*_ mode of W_x_Mo_2x_S_y_ (≈ 410 cm^−1^) is shifted by 8 cm^−1^ to the low‐energy direction, and the $E_{2g}^{1}$ mode of the WS_2_‐like (≈358 cm^−1^) is shifted by 2 cm^−1^ to the high‐energy direction, resulting in a weaker separation of the plane vibration characteristic peak signal modes (≈52 cm^−1^) than that of WS_2_ (≈62 cm^−1^). Among them, the redshift of the *A*_1*g*_ mode of W_x_Mo_2x_S_y_ may be due to the difference in the bonding state of S atoms from that in MoS_2_/WS_2_.^[^ ^47^ ^]^ The $E_{2g}^{1}$ mode redshift of MoS_2_‐like may be due to a large number of W atoms and the $E_{2g}^{1}$ mode blue shift of WS_2_‐like may be due to the small amount of Mo atoms.^[^ ^48^ ^]^ The $E_{2g}^{1}$ mode redshift of MoS_2_‐like is shorter than the blue shift of WS_2_‐like, indicating that most of the atoms of W_x_Mo_2x_S_y_ are still Mo and the doping or alloying of W atoms has resulted in the broadening of the characteristic peaks. The *A*_1*g*_ mode at about 410 cm^−1^ indicates that the thin‐film material synthesized under laser irradiation is an alloyed material W_x_Mo_2x_S_y_, and is not a mixture of MoS_2_/WS_2_.^[^ ^49^ ^]^ The *A*_1*g*_ mode of the Raman spectrum of the MoS_2_/WS_2_ mixture consists of two characteristic peaks, the 408 cm^−1^ for the MoS_2_‐type and the 418 cm^−1^ for the WS_2_‐type. The intervention time of the laser pulse after the addition of the temporally shaped module expands to 10^−15^–10^−12^, and the Raman spectra indicate that the introduction of sub‐pulses does not disrupt the crystallinity of the material. Relative to the material synthesized without the addition of the temporally shaped module (W_x_Mo_2x_S_y_ s‐fs laser), the material synthesized after the introduction of the temporally shaped (W_x_Mo_2x_S_y_ t‐fs laser) introduces more vibrational modes of 130, 228, and 330 cm^−1^, which correspond to the J_1_, J_2_, and J_3_ peaks of the 1T metal phase,^[^ ^50^, ^51^, ^52^ ^]^ indicating that the temporally shaped module is introduced to promote the synthesis of the 1T phase W_x_Mo_2x_S_y_.

Further, we investigated the evolution of the sub‐pulse delay in the W_x_Mo_2x_S_y_ t‐fs laser temporally shaped module as shown in Figure 5b, which shows that the intensities of the characteristic peaks of J_1_, J_2_, and J_3_ covered by the vertical yellow areas, symbolizing the 1T metal phase.^[^ ^50^ ^]^ The intensities of the planar vibrational modes $E_{2g}^{1}$ and *A*_1*g*_ show a tendency to strengthen first and then weaken linearly. The *J*_1_, *J*_2_, and *J*_3_ characteristic peaks symbolizing the 1T metal phase reach the strongest at a delay time of 1 ps when the $E_{2g}^{1}$ and *A*_1*g*_ intensities are the weakest, indicating that the pulse delay of 1 ps is the most favorable for intervening in the chemical reaction changes to affect the crystal structure of the material during the synthesis process. As the fluence increases, the characteristic peak of the alloy phase (358 cm^−1^) increases first and then decreases, achieving the maximum at 4.35J m^−2^ (Figure S13, Supporting Information). The X‐ray photoelectron spectroscopy of the materials synthesized at this parameter was tested as in Figure 5d–f. The peaks of Mo 3d_3/2_ and Mo 3d_5/2_ of 1T W_x_Mo_2x_S_y_ were shifted by 0.9 eV relative to 2H W_x_Mo_2x_S_y_ toward low binding energy, and the peaks of S 2p_1/2_ and S 2p_3/2_ were shifted by about 0.9 eV relative to 2H W_x_Mo_2x_S_y_ toward low binding energy.^[^ ^49^ ^]^ The peaks of W 4f_7/2_ and W 4f_5/2_ are shifted by about 1.0 eV toward the lower binding energy relative to 2H W_x_Mo_2x_S_y_.^[^ ^53^ ^]^ It can be seen that the material synthesized under the 1 ps pulse delay consists of a combination of the 2H and 1T phases. In addition, the Mo 3d pattern contains a pair of double peaks with electronic binding energies of 230.4 eV/233.6 eV, which corresponds to the Mo^5+^ 3d composition, suggesting that the alloyed W_x_Mo_2x_S_y_ undergoes a lattice distortion concerning MoS_2_ and the presence of Mo^V^ defect centers.^[^ ^54^ ^]^ The peak of the Mo 3d pattern located at 230.9 eV corresponds to the Mo^0^ 3d_3/2_, which suggests the W–Mo alloying presence.^[^ ^55^, ^56^ ^]^ The peak of S 2p mapping located at 164.9 eV corresponds to S^0^ 2p, confirming the presence of S in the synthesized product.^[^ ^57^ ^]^ The presence of the S^6+^ 2p peak and W^6+^ 5p peak indicates the occurrence of a small amount of oxidation.^[^ ^58^ ^]^ The surface morphology of the synthesized W_x_Mo_2x_S_y_ films is characterized in Figure 5c, which demonstrates that the films possess clean and atomically flat surfaces. From the XPS total spectrum (Figure S14, Supporting Information), it can be seen that the peak intensities of the W, Mo characteristic peaks in the W_x_Mo_2x_S_y_ synthesized by the temporally shaped high repetition rate femtosecond laser are significantly stronger than those of the hybrid precursor before irradiation. The intensities of the S characteristic peaks are obviously decreased, which suggests that the proportion of S decreases dramatically in the photochemical synthesis process. It is speculated that the S^0^ element generated in the photochemical reaction were vaporized in the transient high‐temperature and high‐pressure environment created by the ultrafast laser. At the same time, the large number of S vacancies generated also contributed to the generation of the 1T phase lattice in W_x_Mo_2x_S_y_ and the alloying of the material. In summary, It was noticed that the high repetition rate temporally shaped photosynthesis system could synthesize high‐quality W_x_Mo_2x_S_y_ thin films with good crystallinity, clean and flat surfaces, and ultra‐wide layer spacing, confirming that the introduction of the temporally shaped module can effectively intervene in the changes of the photochemical reaction to achieve the modulation of the product's lattice structure and synthesize the thin‐film materials with low diffusion energy barriers and high ion‐transport efficiencies.

### 2.4 Electrochemical Performance of the W_x_Mo_2x_S_y_ Alloys MSCs and As‐Prepared TMDs MSCs

**Figure 6:**
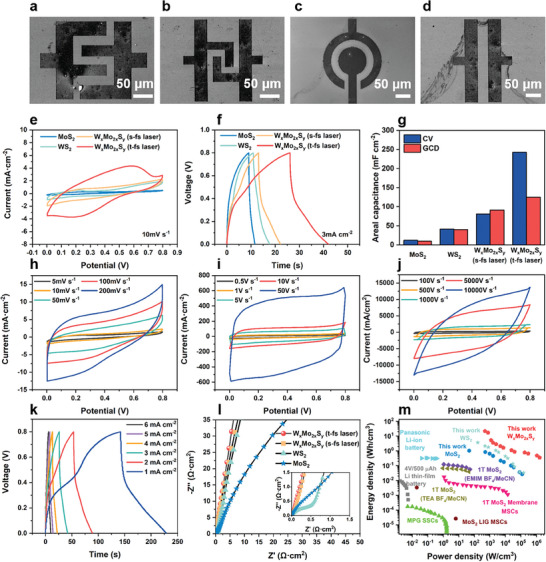
The electrochemical performance of as‐prepared W_x_Mo_2x_S_y_ films. a–d) Scanning electron micrographs of patterned W_x_Mo_2x_S_y_ MSCs with different shapes (interdigital, concentric circle, and parallel strip). e,f) Cyclic voltammetry (CV) and galvanostatic–discharge (GCD) of different supercapacitors anodes synthesized by t‐fs laser photonic‐reduction at 10 mV s^−1^ and 3 mA cm^−2^. g) The area‐specific capacitance of different supercapacitors anodes synthesized by t‐fs laser photonic‐reduction by the CV curves and GCD profiles. h–j) CV curves of the t‐fs laser‐synthesized W_x_Mo_2x_S_y_ with delay time = 1 ps. k) GCD profiles of the t‐fs laser‐synthesized W_x_Mo_2x_S_y_ with delay time = 1 ps at a current density from 1 to 6 mA cm^−2^ under a 0.8 V voltage window. l) Nyquist plots of the different supercapacitor anodes synthesized by t‐fs laser photonic‐reduction. m) Ragone plots of t‐fs‐MoS_2_, WS_2,_ and W_x_Mo_2x_S_y_ supercapacitors, and comparison to other TMDs and thin‐film supercapacitors.

The high repetition rate temporally shaped photosynthesis system enables the high‐quality and flexible synthesis of one‐step patterned thin films, which is of great significance in the field of microenergy storage devices. The W_x_Mo_2x_S_y_ films we synthesized have an ultra‐wide interlayer and a low H‐ion diffusion energy barrier, which are of interest for electrode materials. Therefore, we used this micro‐synthesis system to obtain various materials and fabricated micro‐supercapacitors with a thickness of 10 µm. The one‐step patterned micro‐supercapacitors with various shapes under different exposure parameters are shown in **Figure** **6**a–d. We chose the interdigital micro‐supercapacitors for the subsequent electrochemical testing as Figure 6e–m. An anti‐interference micromanipulation probe bench‐electrochemical workstation test system with signal filtering was used to characterize the electrochemical properties of the devices. The electrochemical performance of various micro‐supercapacitors (MSCs) with the size of 100 µm × 100 µm was contrasted in 1 m H_2_SO_4_. Figure 6e shows the cyclic voltammetry (CV) curves of MSCs prepared by this synthesis system for four material systems (MoS_2_, WS_2_, W_x_Mo_2x_S_y_ s‐fs laser, W_x_Mo_2x_S_y_ t‐fs laser) at a scan rate of 10 mV s^−1^, which can be seen that their shapes are approximate to parallelograms, with obvious capacitance characteristics. The capacitive performance of W_x_Mo_2x_S_y_ is significantly better than that of WS_2_ and MoS_2_, and the electrochemical performance of W_x_Mo_2x_S_y_ synthesized with the modulation of the temporally shaped module is better than that of W_x_Mo_2x_S_y_ synthesized without temporally shaped module. It is attributed to the fact that the spacing of the synthesized interlayers of the W_x_Mo_2x_S_y_ has been greatly widened after the modulation. Meanwhile, it possesses more metal‐phase 1T structures with better electrical conductivity. We varied the pulse delay (0 ps‐10 ps) and energy density (1.63 J m^−2^–5.44 J m^−2^) of the temporally shaped laser to investigate the difference in electrical conductivity of the synthesized W_x_Mo_2x_S_y_ electrode. The electrical conductivity of the material reached its maximum value at a pulse delay of 1 ps and a laser energy density of 4.35 J m^−2^ (Figure S15, Supporting Information). Figure 6f shows the galvanostatic–discharge (GCD) curves at a current density of 3 mA cm^−2^. The constant‐current charge/discharge curves of the four materials are approximately triangular. The W_x_Mo_2x_S_y_ t‐fs laser possesses a higher area‐ratio capacitance at the same current sweep rate, which is further confirmed by Figure 6g. The CV contrast curves of MoS_2_, WS_2_, W_x_Mo_2x_S_y_ s‐fs laser, and W_x_Mo_2x_S_y_ t‐fs laser MSCs at 50 and 100 mV s^−1^ sweep speeds are shown in Figure S16a,b (Supporting Information), further indicating that the W_x_Mo_2x_S_y_ t‐fs laser MSC performs the best. W_x_Mo_2x_S_y_ t‐fs laser MSC has the largest layer spacing and the smallest diffusion barrier for H ions, which is conducive to the improvement of the ion transfer efficiency during charging and discharging, leading to a fast completion of energy storage. Figure 6l depicts the Nyquist plots of MoS_2_, WS_2_, W_x_Mo_2x_S_y_ s‐fs laser, and W_x_Mo_2x_S_y_ t‐fs laser MSCs. The circular regions in the high and mid‐frequency regions characterize the charge transfer impedance of the materials. It can be seen that the W_x_Mo_2x_S_y_ t‐fs laser MSC has a lower charge transfer impedance than the others, and the angle of the spectral lines in the mid‐frequency region of all MSCs is greater than 45° to the real axis, representing the rapid diffusion of ions to the electrodes. The spectral lines in the low‐frequency region are approximately perpendicular, indicating that the four materials have an ideal electrochemical capacitance, with the W_x_Mo_2x_S_y_ t‐fs laser MSC having the most favorable performance. The synthesized MoS_2_, WS_2_, W_x_Mo_2x_S_y_ s‐fs laser, and W_x_Mo_2x_S_y_ t‐fs laser have ultrathin electrode layers and lattice modulation, which results in their very small impedance *R*_ct_ of 0.2631, 0.5792, 0.2369, and 0.08141 Ω, respectively. We performed the electrochemical capacitance of the W_x_Mo_2x_S_y_ (*x* = 1, *y* = 1.5) MSC in the range of 5 mV s^−1^–10000 V s^−1^, and the area‐specific capacitance is calculated to be 242.57mF cm^−2^ and the volume‐specific capacitance is calculated to be 242567 F cm^−3^ at the lowest sweep speed of 5 mV s^−1^. The shape of the probes test curves is shown in Figure S17 (Supporting Information). The capacitance characteristic shapes of the CV curves can be maintained even at very large sweep speeds, in which the CV curve still maintains a certain characteristic shape of capacitance. The Bode plots of MoS_2_, WS_2_, W_x_Mo_2x_S_y_ s‐fs laser, and W_x_Mo_2x_S_y_ t‐fs laser MSCs are shown in Figure S16 (Supporting Information). Since the thicknesses of the synthesized materials are all controlled to be around 10 nm, resulting in the thin‐layer materials and the electrolyte solution being in full contact. We could observe that the frequencies at 45° are very close to each other. The frequency is very close to about 17770 Hz, which translates to a time constant (*τ*_0_) of about 0.056 ms, indicating that the electrode materials have superior multiplicative properties and capacitive behavior. We also established the comparative volume‐specific capacitance of these four materials in Figure S16d (Supporting Information). These results demonstrate the excellent electrochemical performance of the single material/alloyed composite MSCs prepared by the high‐frequency temporally shaped photosynthesis system, with the best performance of the W_x_Mo_2x_S_y_ t‐fs laser.

The synthesis of patterned films made the MSCs preparation simple and flexible. We made three different shapes of MSCs using the W_x_Mo_2x_S_y_ t‐fs laser system to compare the electrochemical performance of interdigital (four‐finger), concentric circle, and parallel strip MSCs. As shown in Figure S18 (Supporting Information), the CV and GCD curves point out the superiority of the interdigital MSCs design. The interdigital MSCs exhibit higher capacitance performance at a current density of 3 mA cm^−1^ because its interdigital structure facilitates the in‐plane contact with the electrolyte and increases the charge transfer sites of the electrodes. The Nyquist and Bode plots show that there is no obvious difference in the overall curves of the three shapes of the MSCs. The interdigital MSCs have smaller impedance *R*_ct_ and larger low‐frequency slopes, indicating that their charge transfer impedance is low and ions diffuse to the electrode material more easily. The frequency at 45° is 25148 Hz, corresponding to a time constant (*τ*_0_) of about 0.040 ms, confirming that the multiplicative performance of the interdigital MSCs is also higher than that of the other MSCs. All tests were performed excluding the effects of the 10 µm probe and substrate (SiO_2_/Si), whose capacitive contributions at the same sweep rate (10 mV s^−1^) were about 14.26 mF cm^−2^ and 7.13 × 10^3^ F cm^−3^, which are much smaller than the capacitive performance of the device. After 8 000 cycles, the MSC retained more than 92.6% of the initial capacitance. The GCD profiles we chose from the cycles were nearly identical under a 0.8 V voltage window, which indicated the stability of the MSCs we prepared (Figure S19, Supporting Information).

We compared the power density and energy density of MoS_2_‐based MSCs from previous work,^[^ ^24^, ^59^, ^60^, ^61^, ^62^ ^]^ plotted as Ragone plots shown in Figure 6m. We compared the electrochemical performance of the synthesized MoS_2_, WS_2_, and W_x_Mo_2x_S_y_ with other devices. The performance of the single material MoS_2_ is orders of magnitude higher than the other capacitors,^[^ ^62^ ^]^ and similar to that of batteries. The WS_2_, as well as the alloyed W_x_Mo_2x_S_y_, even reached unprecedented energy densities. Where the WMo_2_S_1.5_ MSC energy density reaches 21.56 Wh cm^−3^ (power density of 485.13 W cm^−3^) for *x* = 1 and *y* = 1.5. The above results confirm the fast ion exchange capability and excellent energy storage capacity between the alloyed composite electrode W_x_Mo_2x_S_y_ electrode material and the electrolytic. The alloying strategy and interlayer expansion are important ways to optimize the electrochemical performance of 2D energy storage materials for ultrathin films. In order to investigate the effect of MSC structure size on capacitance performance, W_x_Mo_2x_S_y_ MSCs with different thickness/finger interval/size were fabricated meticulously. The trends of the capacitance performance of MSCs with film thicknesses of 10, 15, 20, and 25 nm were investigated by controlling the variables (Figure S20, Supporting Information), and the ratio of the finger intervals of 1, 2, 3, and 5 µm to the MSC capacitance (Figure S21, Supporting Information), and analyzed the effect of overall device size (area) on MSC capacitance performance (Figure S22, Supporting Information). We find that these electrode finger structure dimensions achieve optimization of the MSC performance by affecting the effective electrode material ratio of the device, and the electrolyte ion transport process. Notably, ultrafast laser high‐precision and flexible processing are good at regulating these structural dimensions, which can improve the ion transfer efficiency while retaining the superior properties of the electrode materials to achieve the optimization of MSCs. The laser‐induced one‐step patterning technology provided an important method for the manufacturing of micro devices, that possess a significant research meaning in the future.

## 3 Conclusion

In this paper, a high‐precision light‐controlled atomic level material patterned growth strategy is proposed to synthesize an alloyed W_x_Mo_2x_S_y_ thin film with ultra‐wide layer spacing (13.2 Å), which is applied in the field of ultrathin micro‐supercapacitors. The prepared W_x_Mo_2x_S_y_ MSC has ultra‐high area‐specific capacitance (242.57 mF cm^−2^) and energy density (21.56 Wh cm^−3^). We introduced a temporally shaped module to extend the photochemical reaction intervention time from nanoseconds to picoseconds or even femtoseconds scale and introduced sub‐pulses to modulate the chemical reaction when it is not completed to realize the crystal structure and phase modulation of the material. A high‐repetition rate temporally shaped flexible photosynthesis system has been established for the one‐step synthesis of patterned crystalline films at room temperature, which has wide material applicability. The contactless and maskless transient high‐temperature and high‐pressure synthesis cavity synthesis makes it suitable for a wide range of substrates. We have also simultaneously introduced alloying, interlayer expansion, and effective strategies to enhance charge transport and ion diffusion in thin films, and achieved the reduction of intrinsic diffusion energy barriers through lattice structure modulation. The fast diffusion kinetics and transport behavior of H^+^ in W_x_Mo_2x_S_y_ electrodes are also confirmed by experiments, tests, and density‐functional theory calculations, which are very meaningful for the preparation and study of aqueous energy storage devices. The synthesis system enables the synthesis of 0D, 1D, and 2D multiscale feature‐patterned films with synthesis cell resolution up to 100 nm. Direct synthesis of thin film arrays is realized on 1 inch wafers, and the unphotolithographed films are continuous, surface clean, and atomically flat. The flexible and controllable photosynthesis method is of great significance for miniaturized and miniaturized fabrication of devices.

## 4 Experimental Section

### (NH_4_)_2_WS_4_/(NH_4_)_2_MoS_4_ Hybrid Precursors Solution Preparation and the Thin Film Spin–Coated on a SiO_2_/Si Wafer

The (NH_4_)_2_MoS_4_ (CAS No. 15060‐55‐6) and (NH_4_)_2_WS_4_ (CAS No. 13862‐78‐7) powder were purchased from Sigma–Aldrich. Dimethylformamide (DMF), n‐butylamine, and 2‐aminoethanol were used as solutions, which were supplied from InnoChem, Ltd. (Beijing, China). They were mixed in a ratio of 5:2:1 (v/v/v) to dissolve the composite precursor powder((NH_4_)_2_MoS_4_:(NH_4_)_2_WS_4_ = 1:1 (m/m))adequately. The hybrid precursor solution was magnetically stirred for 1 h and then sonicated for 30 min at 50 °C. The substrates (SiO_2_/Si wafer, dielectric layer 300 nm) were cleaned by acetone, ethanol, and deionized water before 30 min treatment by oxygen plasma. The hybrid precursor was then uniformly spina‐coated onto the substrate with the speed of 500r s^−1^ for 30 s and 2000r s^−1^ for 1 min.

### Selective Photonic Reduction by the Temporally Shaped High‐Repetition‐Rate Femtosecond Laser

A Ti: sapphire femtosecond laser oscillator (wavelength = 800 nm, pulse width = 35 ps, and repetition rate = 84 MHz) was used to induce a local high‐repetition‐rate photonic‐reduction reaction. A beam splitter was used at the beginning of the temporal pulse train. The pulse train was focused using a 20× objective lens (Olympus, NA = 0.45). The sample was horizontally placed on a six‐axis translation stage (M840.5DG, PI, Inc.). The patterned path was defined precisely via G‐code translated from a preset pattern by graphic design software(Autodesk ArtCAM 2019). Definition of patterning line spacing based on focused laser energy density and path travel velocity.

### Characterization of Laser‐Induced Transition Metal Dichalcogenide and W_x_Mo_2x_S_y_ Alloys

The morphology of the film was characterized by an optical microscope (OM) using an Olympus metallographic microscope. The micron and submicron feature patterns were captured by a cold field emission scanning electron microscope (SEM) using Regulus 8230 (Hitachi, Japan). The thickness of films was tested by an atomic force microscope (AFM) using Bruker Dimension Fastscan (Bruker, German) at Tsinghua University. An InVia Reflex spectrometer (Renishaw, UK) was used to harvest Raman spectroscopy with the excitation laser line at 532 nm. An Escalab Xi+ spectrometer (ThermoFisher Scientific, UK) with a monochromatic Al Kα source (50 µm beam spot) was employed to investigate the X‐ray photoelectron spectroscopy (XPS). The electrochemical performance was measured by an electrochemical workstation using CHI760E (Chenhua, China).

### Theoretical Calculations

The electronic structure calculations and diffusion barrier calculations were proposed to analyze the physical and chemical properties of materials using the Vienna ab initio simulation package (VASP) code.^[^ ^63^ ^]^ The Perdew–Burke–Ernzerhof (PBE) function was adopted to implement in electronic structure calculations. The cutoff energy of 540 eV was adopted in structural optimization. A 4 × 4 supercell was used to study the effect of interlayer expansion of WMo_2_S_1.5_ on the diffusion barrier and calculate the density of states of 2H MoS_2_ and as‐synthetized WMo_2_S_1.5_. Experimental lattice constants for the WMo_2_S_1.5_ (*a* = 1.7660 nm, *c* = 2.3491 nm, *α* = 90.57°, and *γ* = 88.40°) and bulk 2H MoS_2_ (*a* = 0.6320 nm, *c* = 1.2294 nm, *α* = 90°, and *γ* = 120°) were used for all.^[^ ^44^, ^45^, ^46^ ^]^ The computationally optimized WMo_2_S_1.5_ interlayer spacing (13.2 Å) fits with the experimental reference values, which indicates the applicability of the chosen computational setup. The quality consistency of WMo_2_S_1.5_ and the comparison results between WMo_2_S_1.5_ and 2H‐MoS_2_ were confirmed many times.

### Electrochemical Characterization of the Result of Micro‐Supercapacitors

The electrochemical performance of W_x_Mo_2x_S_y_ was measured by a CHI760E electrochemical workstation connected to a Probe Station with gold‐coated tungsten steel probes (tip diameter, ≈10 µm) as the current collectors. The measurement was carried out in a two‐electrode system. The open‐circuit potential (*E*_ocp_) tests were implemented one hour before every measurement to ensure a stable electrochemistry environment. The main electrochemical characterization was based on CV, galvanostatic charge/discharge (GCD), and electrochemical impedance spectra (EIS). The areal capacitance (mF cm^−2^) and volumetric capacitance (mF cm^−3^) per electrode were derived from the CV and GCD tests using Equations. (2) and (3), respectively, as follows:

(2) $$C=\frac{1}{2\vartheta \times V}\int \begin{array}{c} V_{f}\\ V_{i} \end{array}I\left(V\right)dV$$

where *I*, *ϑ*, and *V* represent the current applied, scanning rate, and voltage (*V*_f_ and *V*_i_ are the final voltage and initial voltage).

(3) $$C=\frac{I}{\left(-dV/dt\right)}$$

where *I* is the discharge current, and dV/dt is the slope of the discharge curve. Cycling stability measurements were performed by repeating constant current charge‐discharge at 2 mA for 10000 cycles. The energy densities (mWh cm^−2^) of the supercapacitors were calculated according to the following equations:

(4) $$E_{cell}=C_{cell}\Delta E^{2}/\left(2\times 3,600\right)$$

where ∆*E* is the operating voltage window. Therefore, the power density (µWh cm^−2^) of the obtained supercapacitor was obtained as follows:

(5) $$P_{cell}=E_{cell}\times 3,600/t$$

where *t* represents the discharge time (*t* = ∆*V*/*ϑ*).

The fact that the size of our capacitor is 100 µm×100 µm. The above electrochemical performance excluded the influence of probes. So we measured the capacitance of electrodes and the tungsten probe together in the same electrolyte. The results were corrected to the true capacitance value *C*_cell_ = *C*_electrodes_−*C*_tungsten probe_, which eliminates interference from the tungsten probe.

## Conflict of Interest

The authors declare no conflict of interest.

## Supporting information

Supporting Information

## Data Availability

The data that support the findings of this study are available in the supplementary material of this article.
