# Supplementary material for: Ultra‐Wide Interlayered WxMo2xSy Alloy Electrode Patterning through High‐Precision Controllable Photonic‐Synthesis
Source: Adv Sci (Weinh). 2024 Jul 29;11(36):2403378. doi: 10.1002/advs.202403378 (PMC11423094; doi:10.1002/advs.202403378)
Supplement: Supplementary file 1 — Supporting Information [file ADVS-11-2403378-s001.docx]

**Supporting Information**

**Ultra-wide Interlayered W_x_Mo_2x_S_y_ Alloy Electrode Patterning through High-** **precision Controllable Photonic-** **synthesis**

*Mengyao Tian^1^, Xin Li^1^, Aisheng Song**^4^, Chenyang Xu^1^, Yongjiu Yuan^1^, Qian Cheng^1^, Pei Zuo^1^, Sumei Wang ^1^, Misheng Liang^1^,* *Ruoxi Wang^1^, Tianbao Ma^4^, Liangti Qu^5^ and Lan Jiang^1,2,3, *^*

^1^ Laser Micro/Nano-Fabrication Laboratory, School of Mechanical Engineering, Beijing Institute of Technology, Beijing 100081, P. R. China

^2^ Yangtze Delta Region Academy of Beijing Institute of Technology, Jiaxing 314019, P.R. China

^3^ Beijing Institute of Technology Chongqing Innovation Center, Chongqing 401120, P. R. China

^4^ State Key Laboratory of Tribology, Tsinghua University, Beijing 100084, P.R. China

^5^ MOE Key Laboratory of Bioorganic Phosphorus Chemistry & Chemical Biology, Department of Chemistry, Tsinghua University, Beijing, P. R. China.

^*^E-mails: jianglan@bit.edu.cn


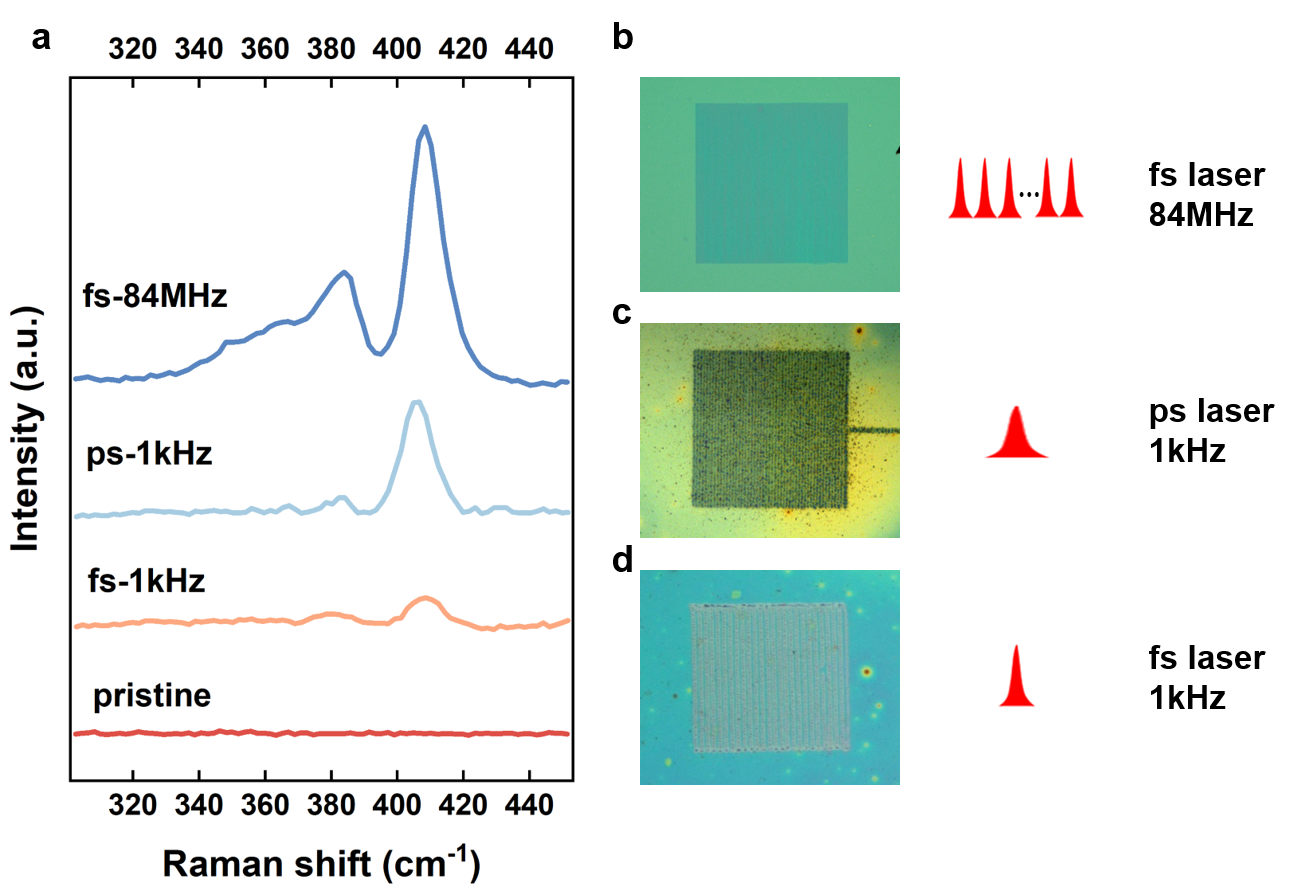


**Figure S1.** Effect of repetition frequency and laser pulse width on material synthesis. (a) Raman spectra contrast of the synthesized alloy W_x_Mo_2x_S_y_ mode among different laser processing. (b-d) The optical images of W_x_Mo_2x_S_y_ films synthesized by femtosecond laser and picosecond laser with different repetition frequency.


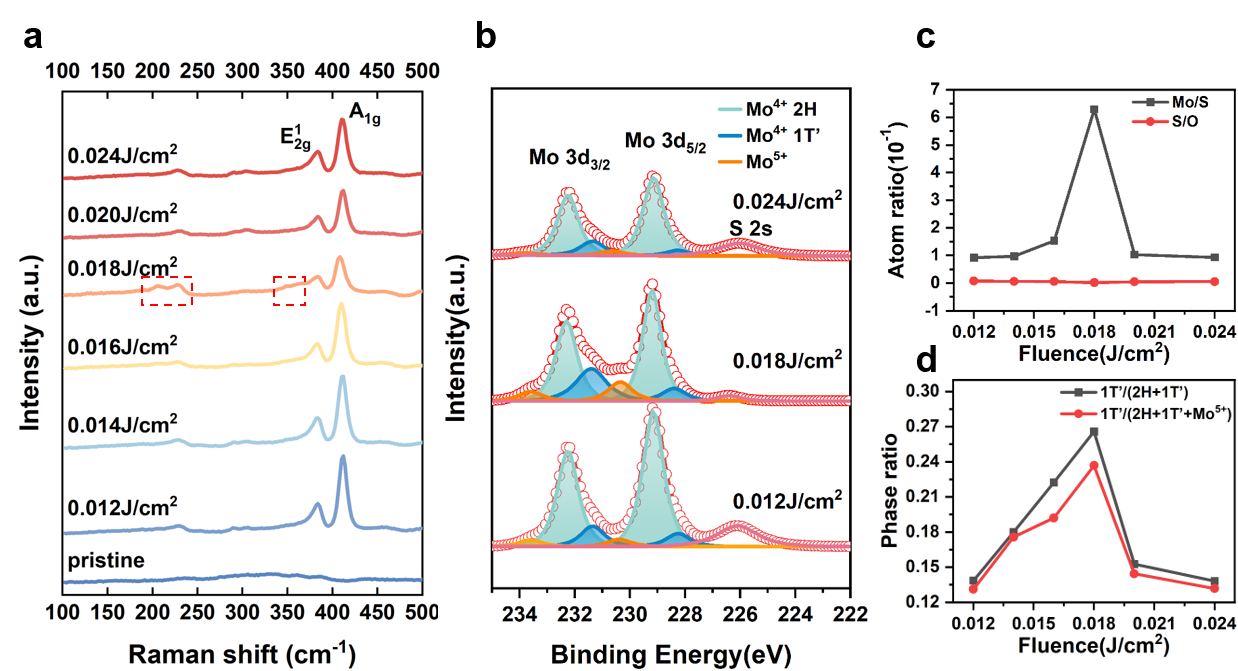


**Figure S2.** Effect of energy density of high repetition frequency femtosecond lasers on lattice vibrations and crystal phase ratio of synthetic MoS_2_ materials. (a) Raman spectra contrast of the synthesized MoS_2_ mode among different fluence of t-fs laser- synthesis processing. (b) XPS spectra (Mo) of the patterned MoS_2_ MSCs. (c-d) Elemental ratios and crystal ratios of synthesised MoS_2_ in relation to the fluence of high repetition frequency femtosecond laser.


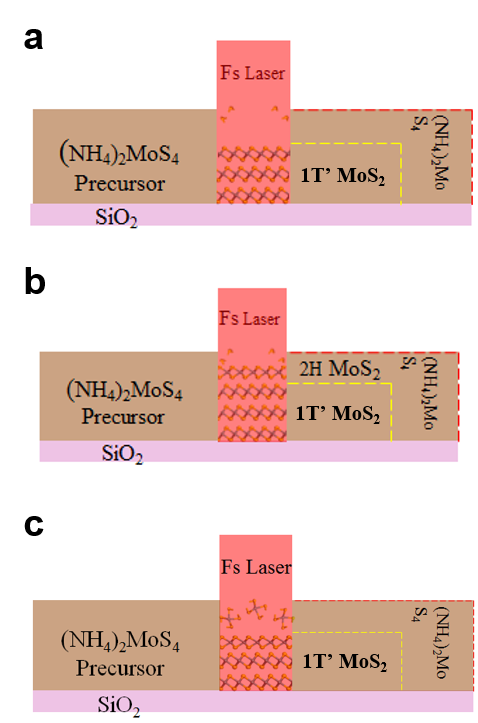


**Figure S3.** Schematic representation of the synthesis-modification-removal of materials induced by laser ablation of the precursor ammonium tetrathiomolybdate in different energy intervals.


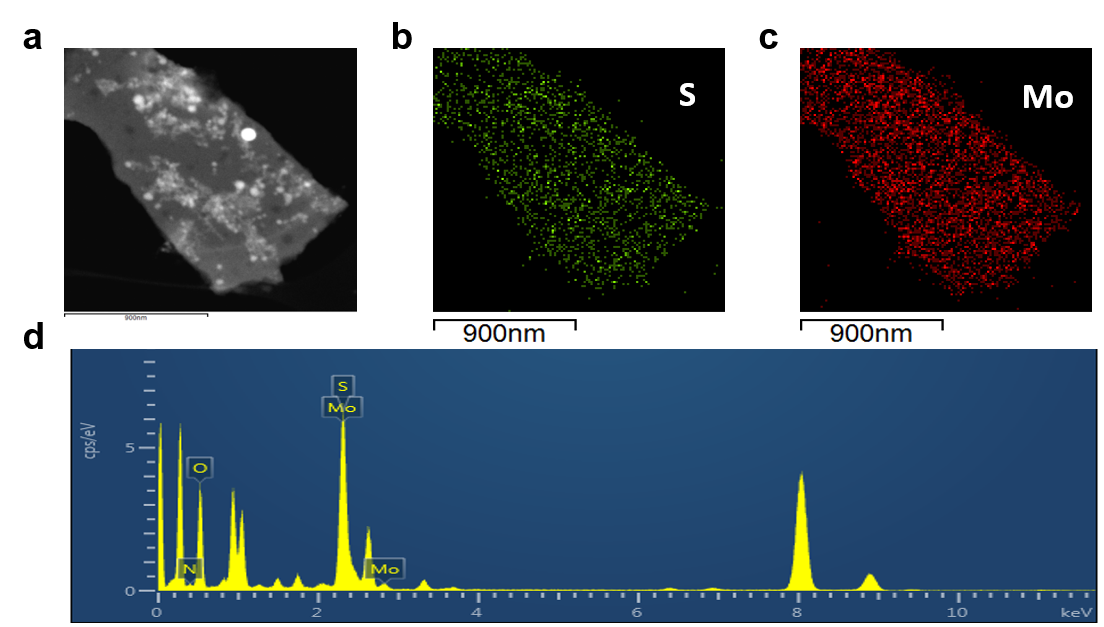


**Figure S4.** Transmission electron microscopy elements (Mo and S) mapping and spectra of as-synthesised MoS_2_.


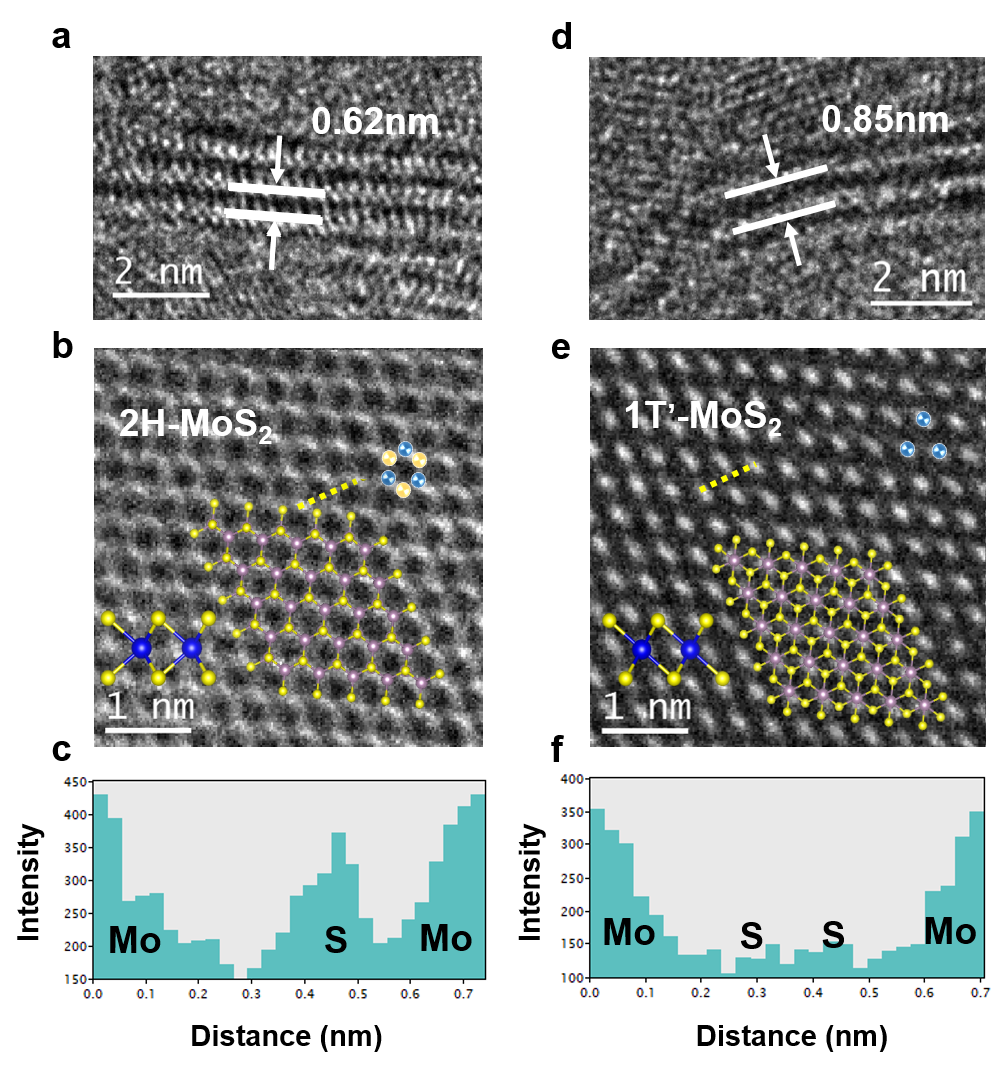


**Figure S5.** The TEM images of 2H and 1T’-phase MoS_2_ with different layer spaces and lattice structures obtained by laser modulation.


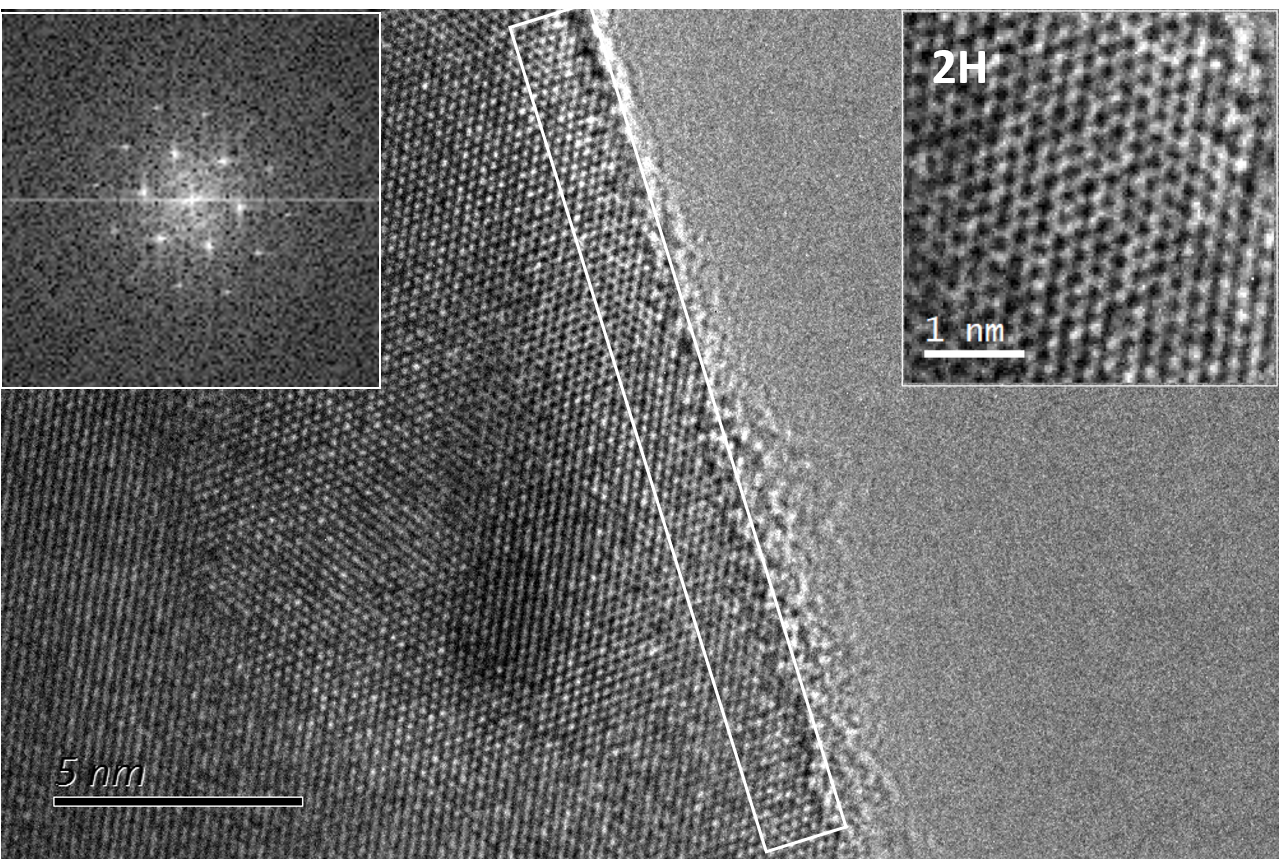


**Figure S6.** The TEM images of 2H phase MoS_2_.


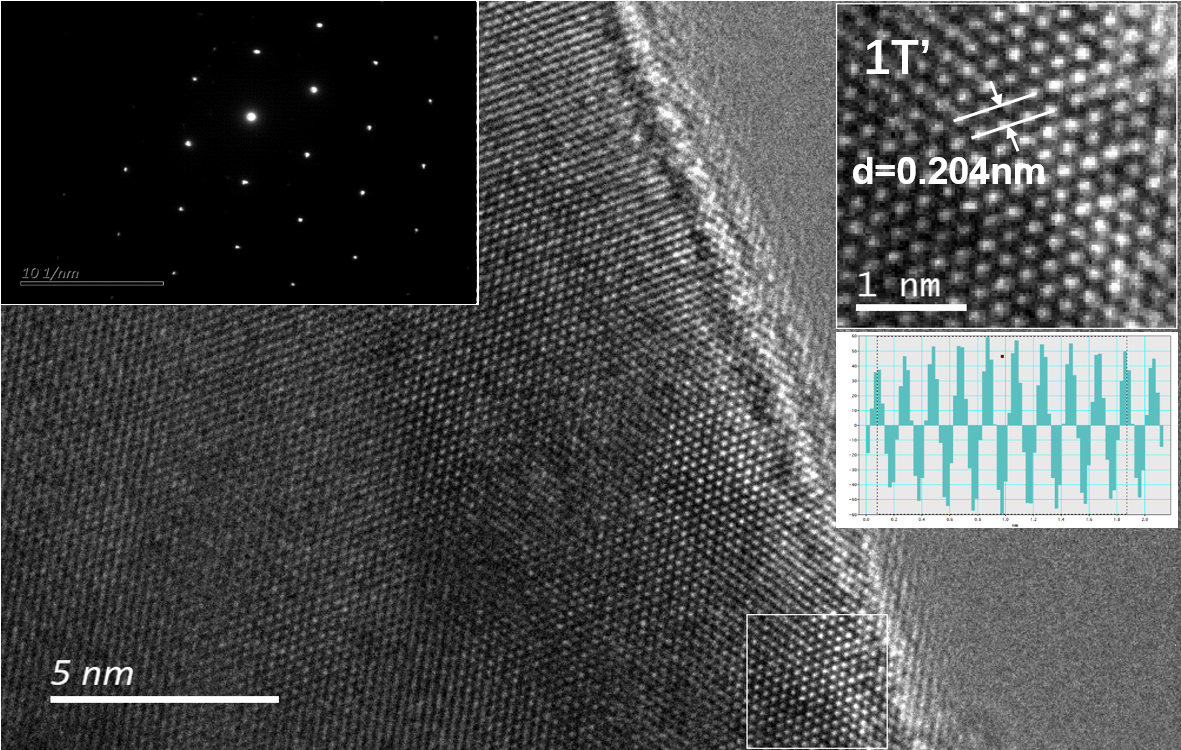


**Figure S7.** The TEM images of 1T’ phase MoS_2_.


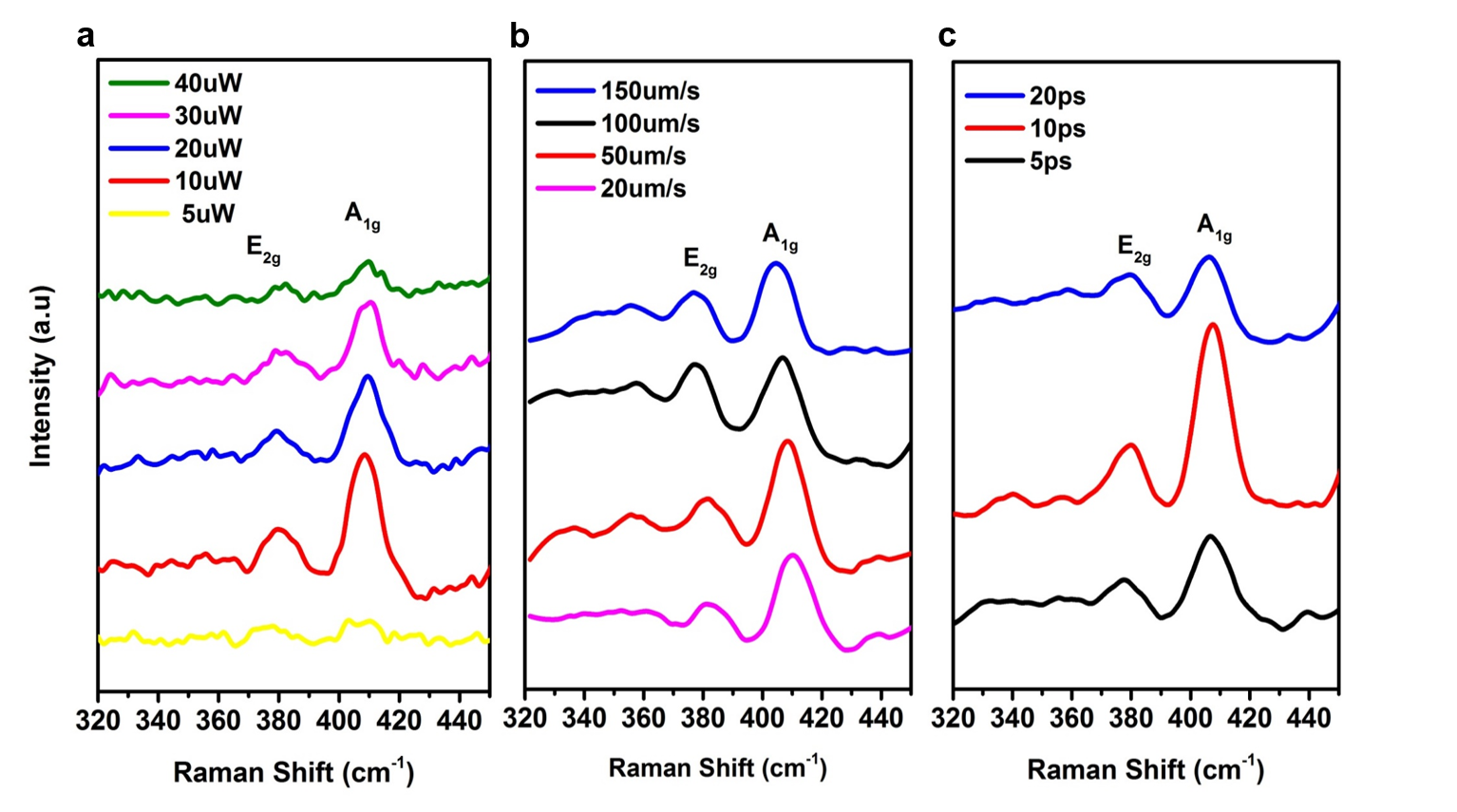


**Figure S8**. The comparison of the synthesized MoS_2_ raman mode among different fluence, scan speed and time-delay of t-fs laser- synthesis processing.


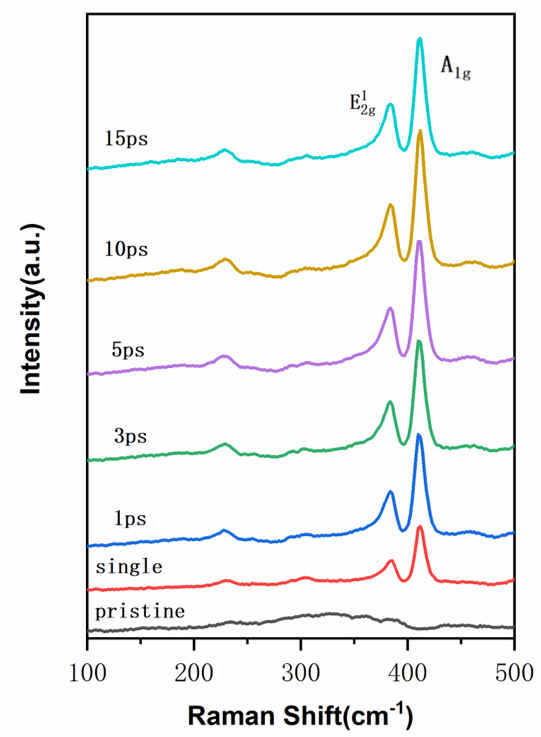


**Figure S9.** Raman spectra contrast of the synthesized MoS_2_ mode among different time-delay of t-fs laser- synthesis processing.


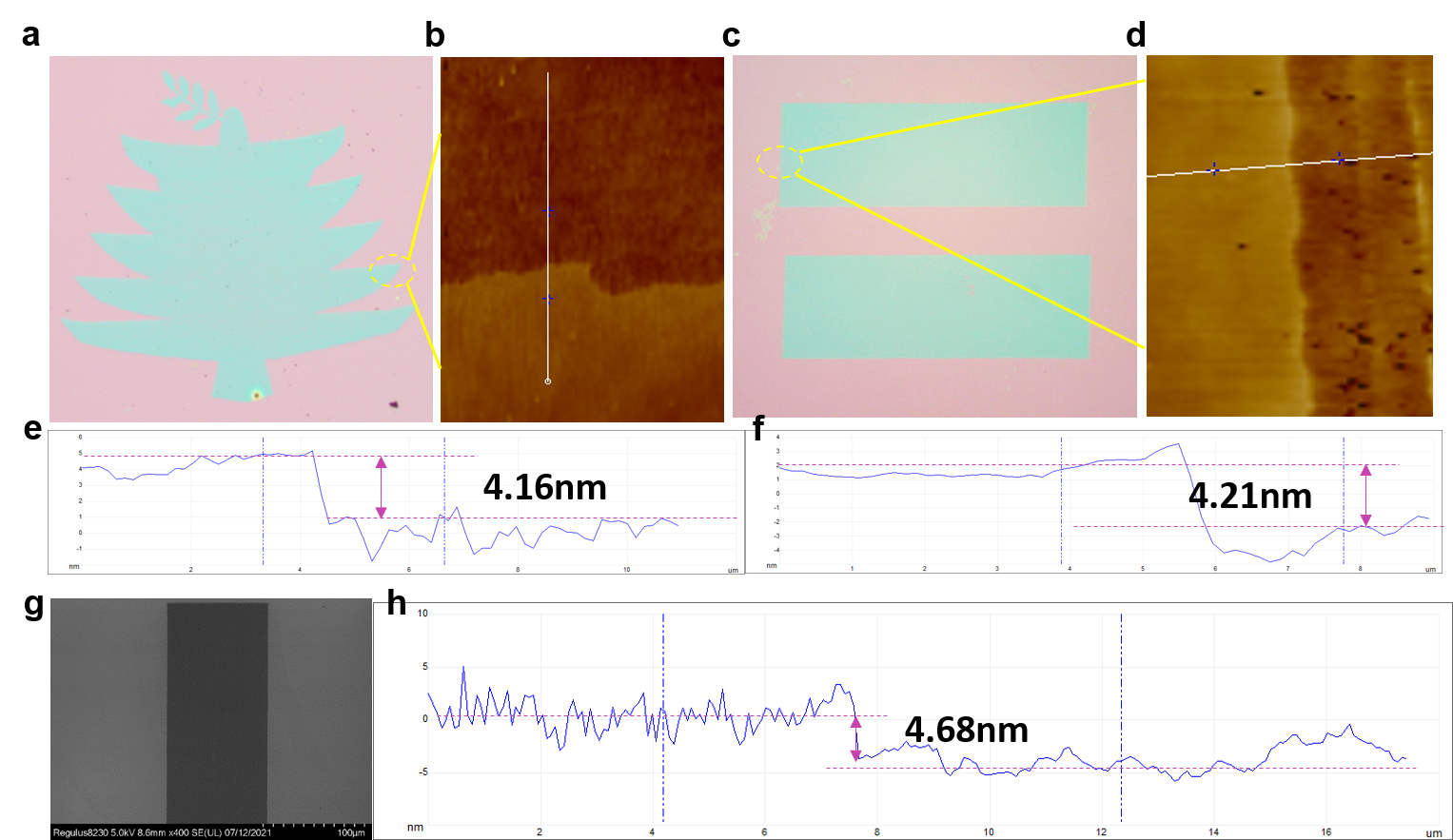


**Figure S10.** Surface morphology and thickness characterisation of MoS_2_ synthesised by high heavy frequency femtosecond laser. (a) and (c) The optical images of as-prepared MoS_2_ film. (b) and (d) The AFM images at the edge magnification. (e-h) The thickness profile of films. (g) The SEM images of MoS2 film in (c). (h) The thickness profile of (g).


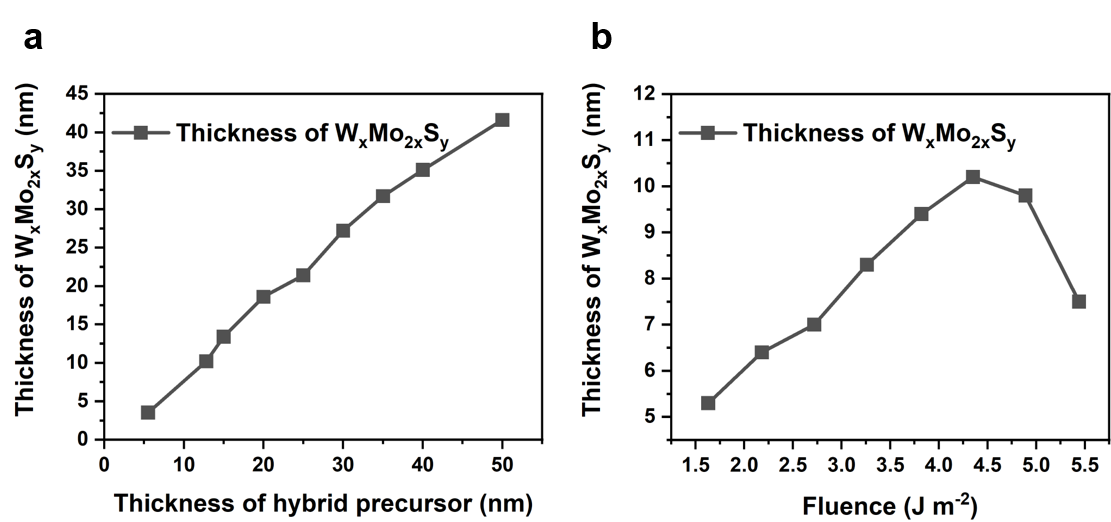


**Figure S11.** The effect of precursor thickness and laser fluence on film thickness (single irradiation molding).


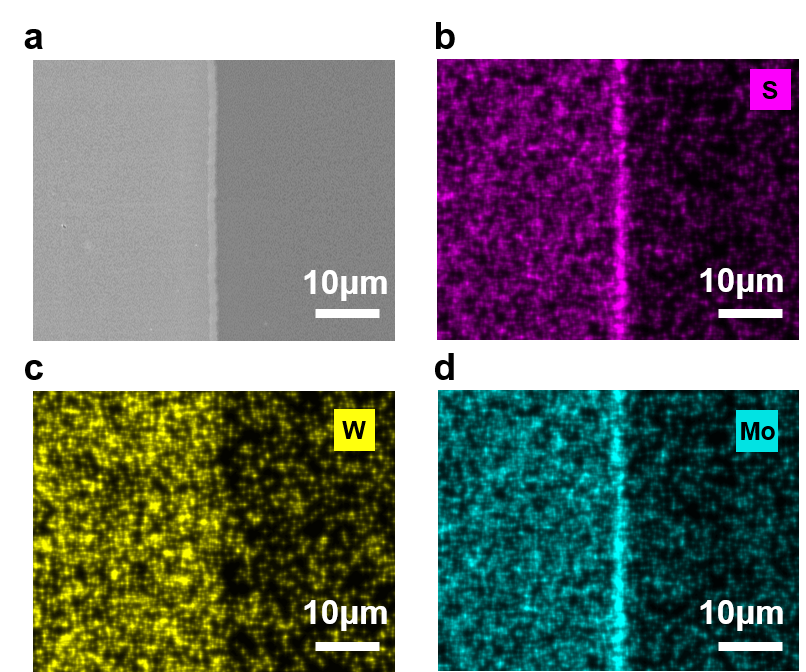


**Figure S12.** Characterization of W_x_Mo_2x_S_y_ film surface morphology and composition. (a) The SEM image of W_x_Mo_2x_S_y_ film on SiO_2_/Si substrate. (b-d) EDS mapping images of the synthesized W_x_Mo_2x_S_y_ film (including Mo, W, S).


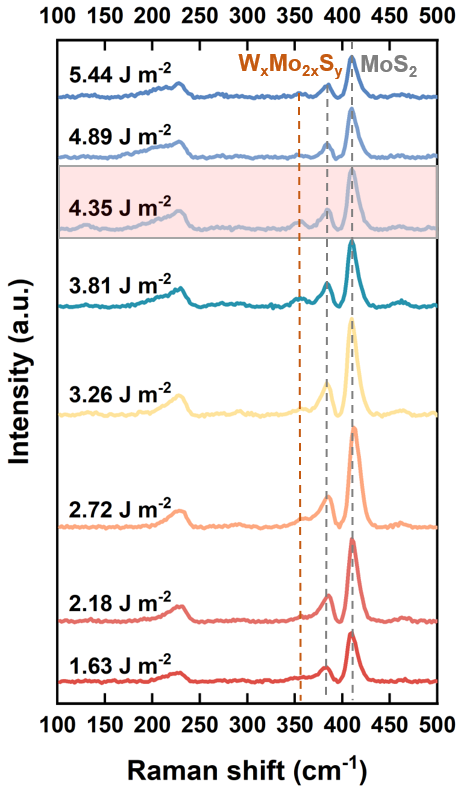


**Figure S13.** Raman spectra contrast of the synthesized alloy W_x_Mo_2x_S_y_ mode among different fluence of t-fs laser- synthesis processing.


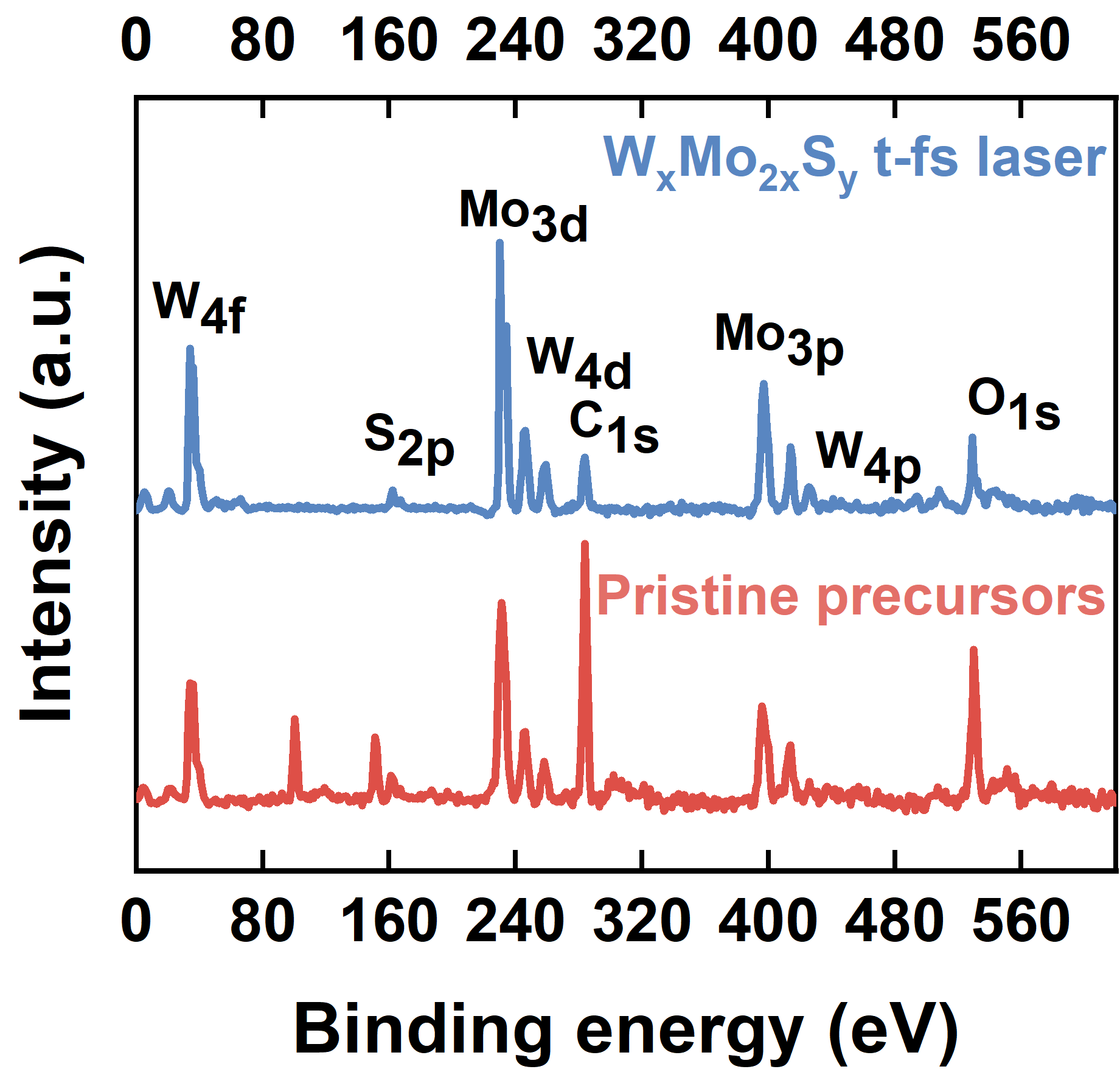


**Figure S14.** XPS total spectrum of the synthesized alloy W_x_Mo_2x_S_y_ (pulse delay=1ps) and pristine mixed precursors.


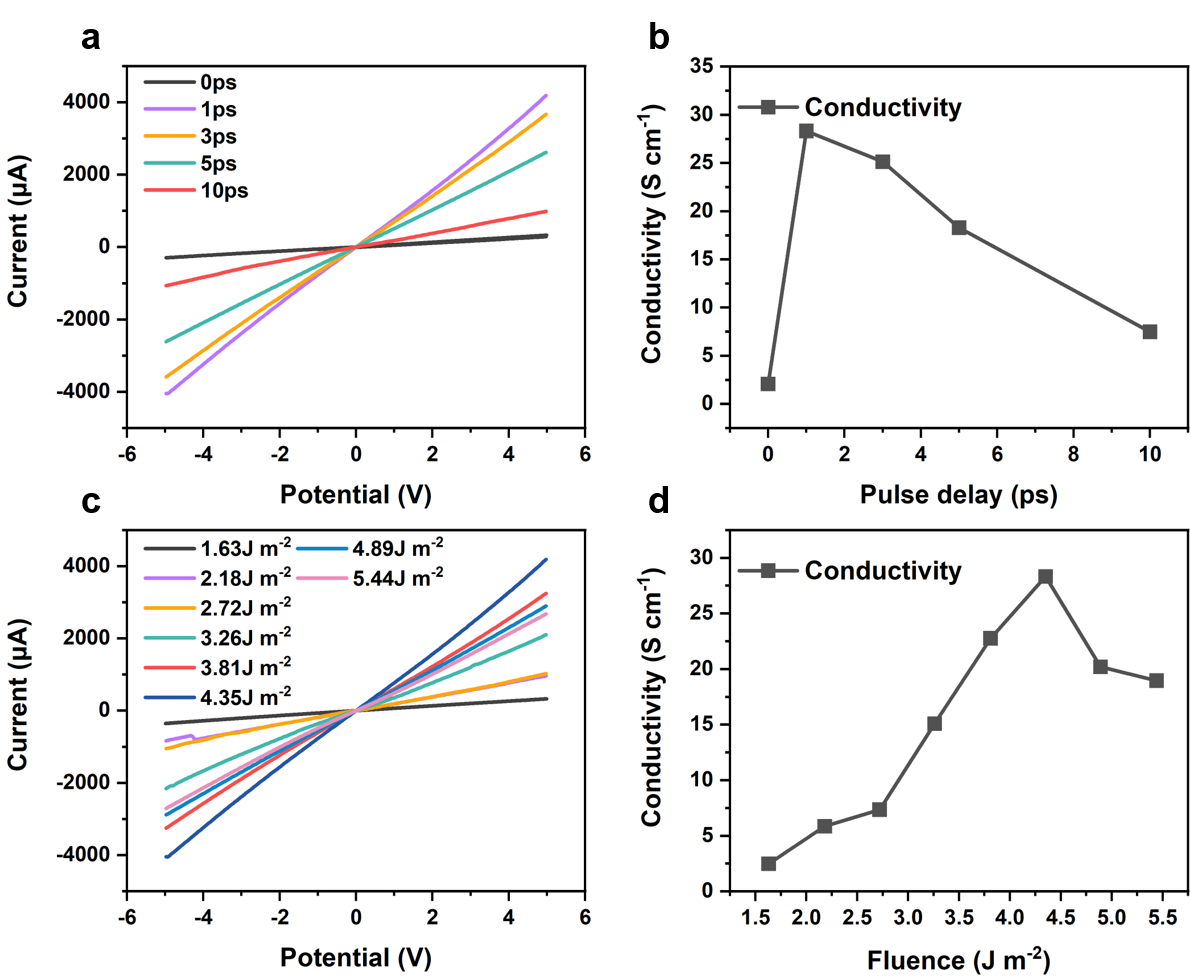


**Figure S15.** The conductivity of as-prepared W_x_Mo_2x_S_y_**.** (a) The resistance of W_x_Mo_2x_S_y_ electrodes induced by different pulse delay at a potential of 5V. (b) The conductivity of the W_x_Mo_2x_S_y_ electrodes changes as the pulse delay time increasing. (c) The resistance of W_x_Mo_2x_S_y_ electrodes induced by different laser fluence at a potential of 5V. (d)The conductivity of the W_x_Mo_2x_S_y_ electrodes changes as the laser fluence increasing.


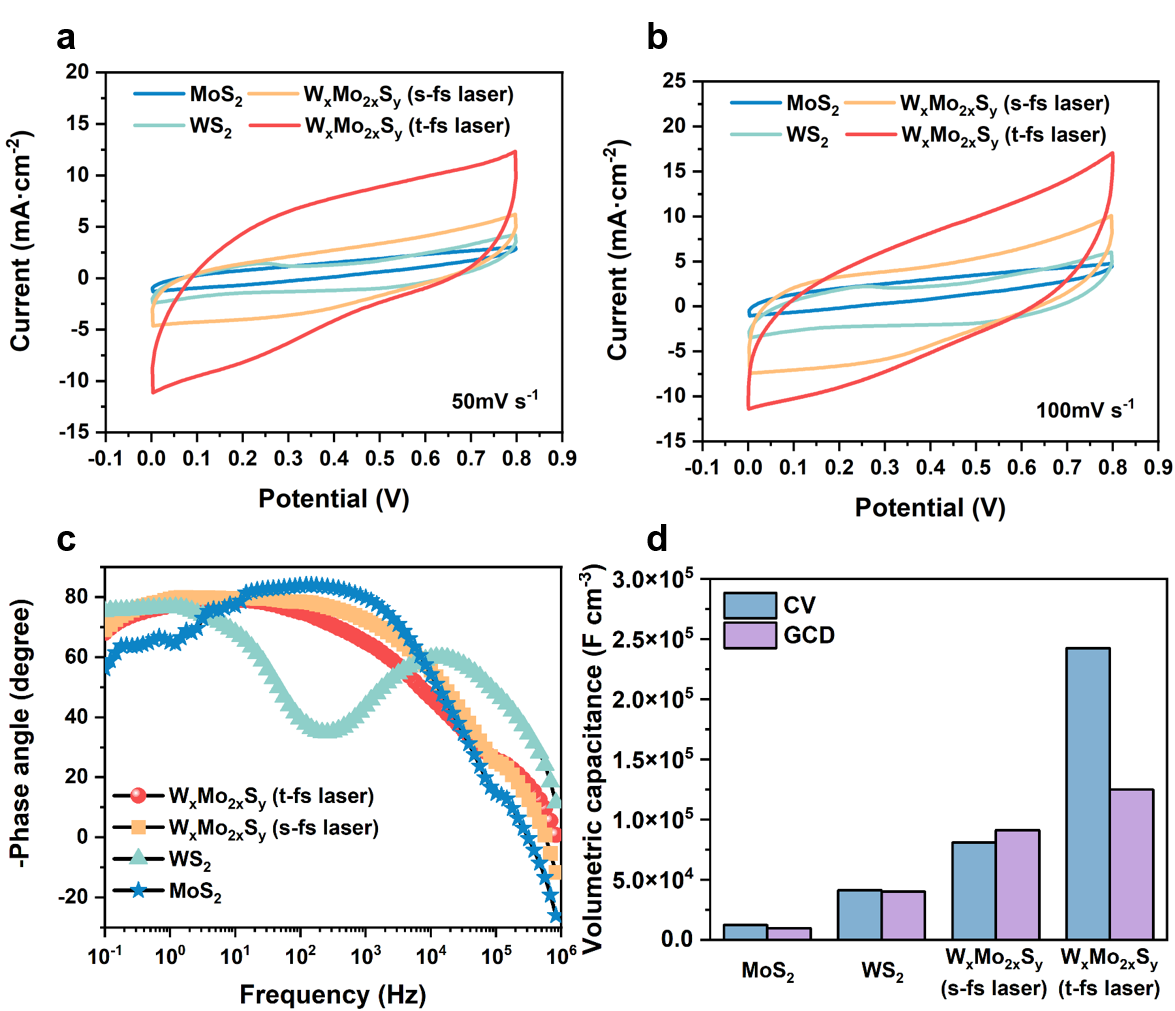


**Figure S16.** The electrochemical performance of as-prepared MSCs in different materials. (a-b) Cyclic voltammetry (CV) of supercapacitor anodes with different materials synthesized by t-fs laser photonic-reduction at 50 mV s^−1^ and 100 mV s^−1^. (c) Bode plots of the different supercapacitor anodes synthesized by t-fs laser photonic-reduction. (d) The volumetric specific capacitance of different MSCs synthesized by t-fs laser photonic-reduction by the CV curves and GCD profiles. (The thickness of tested films is10nm.)


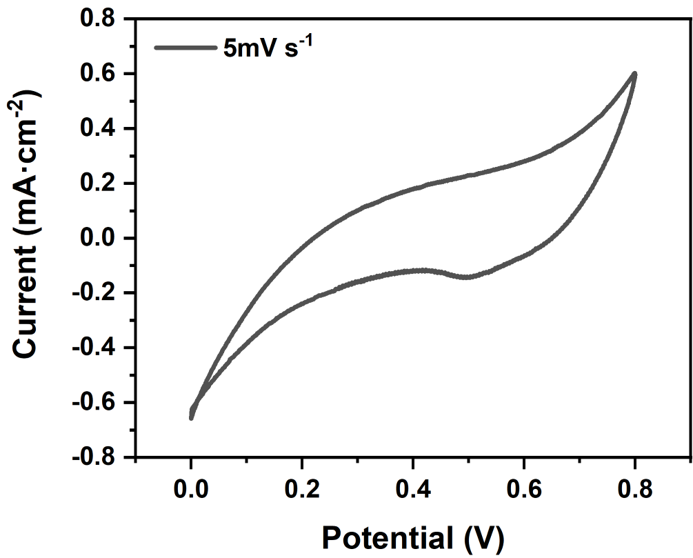


**Figure S17.** Cyclic voltammetry (CV) of the 10μm probes we used for the electrochemical test at 5 mV s^−1^.


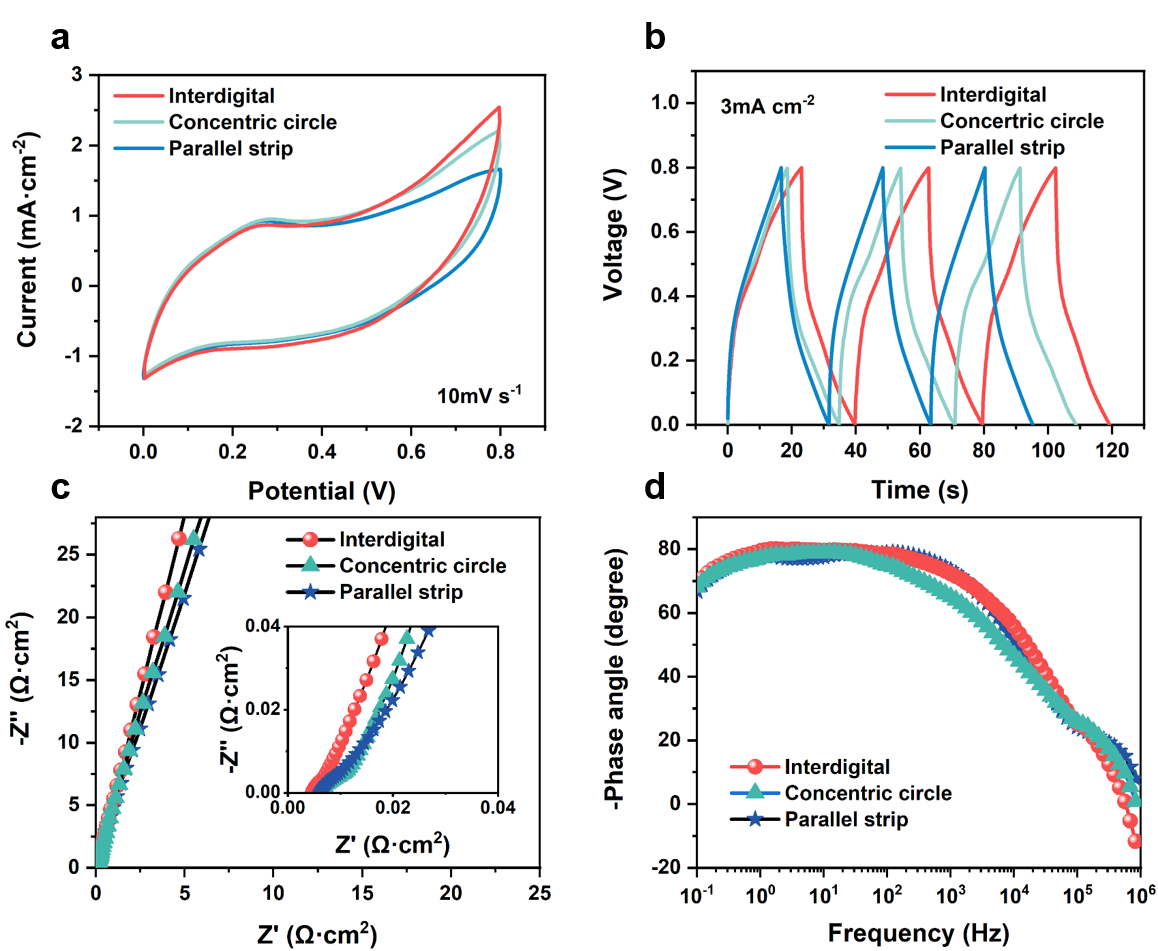


**Figure S18.** The electrochemical performance of as-prepared MSCs in different shapes. (a) Cyclic voltammetry (CV) of MSCs in different shapes synthesized by t-fs laser photonic-reduction at 10 mV s^−1^. (b) Galvanostatic charge–discharge (GCD) of MSCs synthesized by t-fs laser photonic-reduction at 3 mA cm^-2^. (c) Nyquist plots of the different supercapacitor anodes synthesized by t-fs laser photonic-reduction. (d) Bode plots of the different supercapacitor anodes synthesized by t-fs laser photonic-reduction.


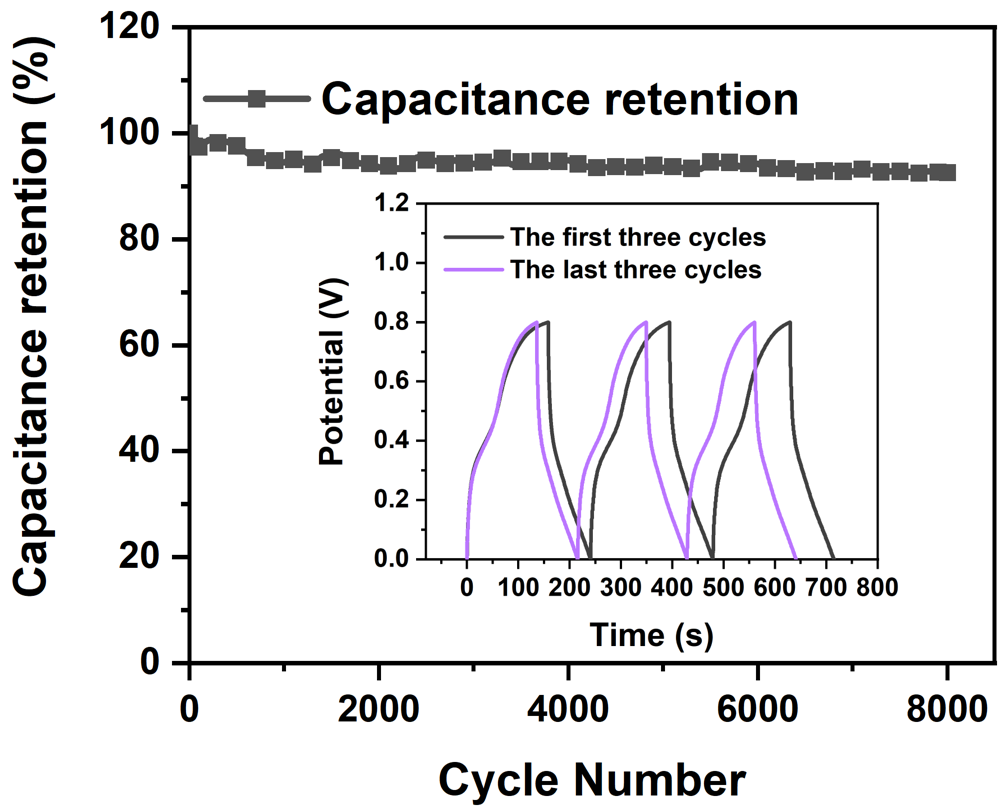


**Figure S19.** The electrochemical stability of as-prepared MSCs in the GCD profiles with a cycle of 8000 under a 0.8 V voltage window.


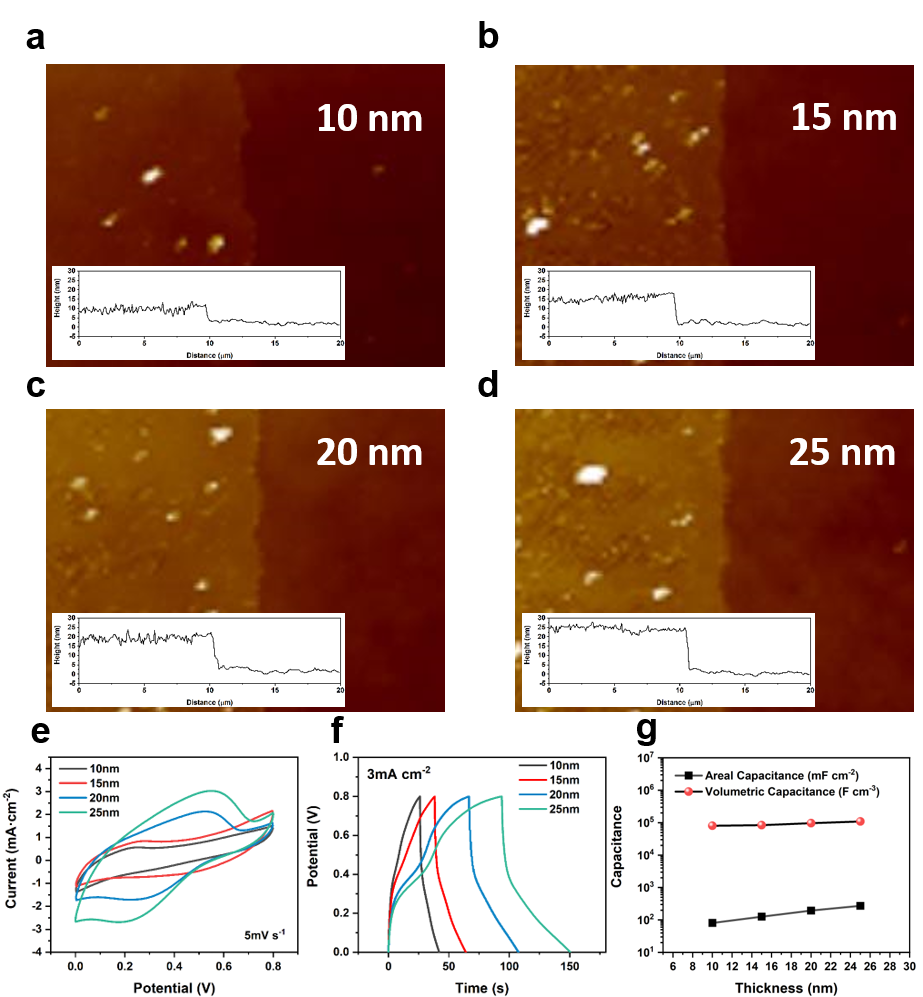


**Figure S20.** Relationship between film thickness and electrochemical properties. (a-d) The atomic force micrographs of few-layer W_x_Mo_2x_S_y_ film with the height of 10nm, 15nm, 20nm and 25nm. CV curves (e) and GCD profiles (f) of MSCs under different film thicknesses were tested, and (g) the areal and volumetric capacitance of MSCs under different thicknesses.


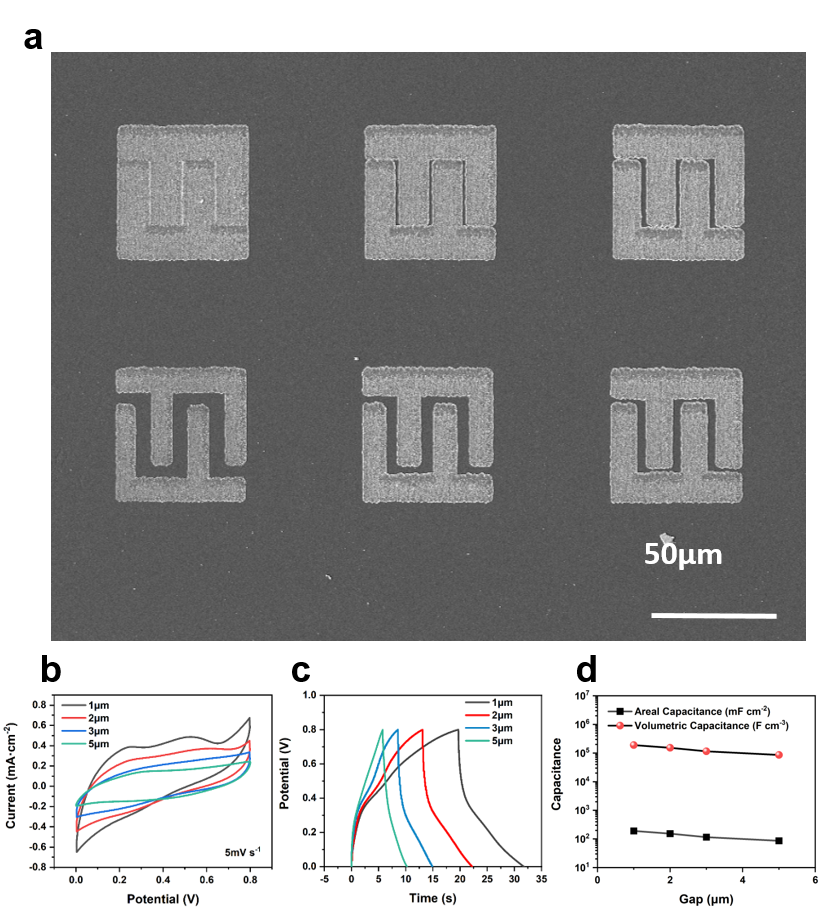


**Figure S21.** Relationship between finger gaps and electrochemical properties. (a) The SEM images of W_x_Mo_2x_S_y_ films with the finger gap of 200nm, 1μm, 2μm, 3μm, 5μm and 8μm. CV curves (a) and GCD profiles (b) of MSCs with different finger gaps were tested, and (c) the areal and volumetric capacitance of MSCs with different finger gaps.


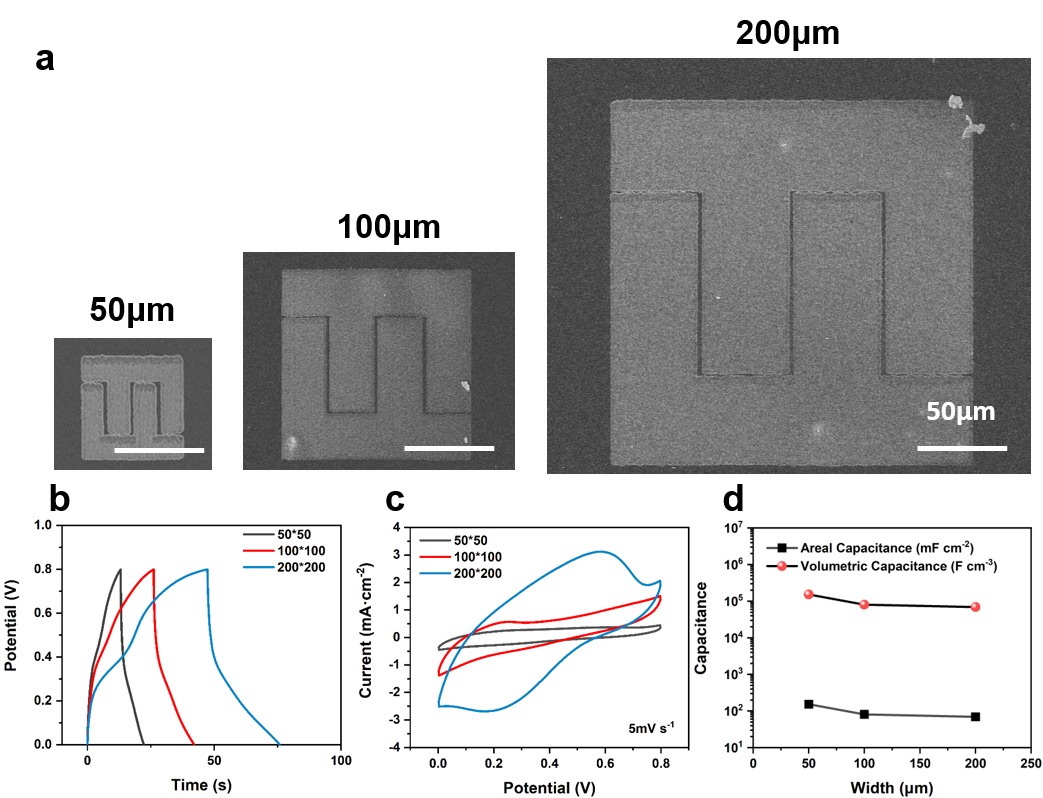


**Figure S22.** Relationship between MSC widths and electrochemical properties. (a) The SEM images of W_x_Mo_2x_S_y_ MSCs with the width of 50μm, 100μm, 200μm. CV curves (a) and GCD profiles (b) of MSCs with different widths were tested, and (c) the areal and volumetric capacitance of MSCs with different widths.

**Table S1 |** The element ratio of alloyed material W_x_Mo_2x_S_y_ through the controllable photonic- synthesis with different time-delay in the fluence of 4.35 J m^-1^.

| **Time delay**  **(ps)** | **Fluence**  **(J m^-1^)** | **W ratio**  **(%)** | **Mo ratio**  **(%)** | **S ratio**  **(%)** | **W:Mo:S** | **Interlayer space (Å)** |
| --- | --- | --- | --- | --- | --- | --- |
| 0 | 4.35 | 22 | 43 | 35 | 1:2:1.6 | 9.5 |
| 1 |  | 24 | 47 | 29 | 1:2:1.5 | 13.2 |
| 3 |  | 21 | 43 | 36 | 1:2:1.7 | 11.3 |
| 5 |  | 21 | 42 | 37 | 1:2:1.8 | 8.9 |
| 10 |  | 20 | 40 | 40 | 1:2:2 | 8.5 |

**Table S2 |** Summary of the film-supercapacitor devices.

| **Electrode**  **material** | **Gel**  **electrolyte** | **Thickness(nm)** | | **Voltage**  **window** | **Capacitance** | **Maximum Power Density** | **Maximum Energy Density** | **Ref.** |
| --- | --- | --- | --- | --- | --- | --- | --- | --- |
| Au nanorime  mesh | PVA-H_2_SO_4_ | 550 | 0.8 V | | 165.4  μF cm^−2^ | 4.04  mW cm^−2^ | 3.80  μWh cm^−2^ | ^[1]^ |
| MnO_2_–Au–Ni | PVA-LiCl | 550 | 0.8 V | | 21.25  mF cm^−2^ | 8.02  mW cm^−2^ | 1.89  μWh cm^−2^ | ^[2]^ |
| Graphene  /PANI | PVA-H_2_SO_4_ | - | 0.8 V | | 95.5 F cm^−3^ | - | - | ^[3]^ |
| 1T’ MoTe_2_ | Na_2_SO_4_ | 5 | 1.0V | | 18700  F cm^−3^ | 100  W cm^−3^ | 1.56  Wh cm^−3^ | ^[4]^ |
| Bio-C/MoS_2_ | Na_2_SO_4_ | - | 0.5V | | 945  F g^−1^ | 8000  W kg^−1^ | 157  Wh kg^-1^ | ^[5]^ |
| 1T-MoS_2_ | H_2_SO_4_ | 1500 | 0.5V | | 437  F cm^−3^ | - | - | ^[6]^ |
| Ni-doped MoS_2_ | PVA-KOH | - | 1.6V | | 110.1  F g^−1^ | 11520  W kg^−1^ | 140.9  Wh kg^-1^ | ^[7]^ |
| Co-EG/SV-MoS_2_ | K_2_SO_4_ | - | 1.0V | | 1884.36 mF cm^−2^ | 0.84  mW cm^−2^ | 0.51  mWh cm^−2^ | ^[8]^ |
| MoS_2_ | H_2_SO_4_ | 10 | 0.8 V | | 12124  F cm^−3^ | 24.2 W cm^−3^ | 1.08  Wh cm^−3^ | This work |
| WS_2_ | H_2_SO_4_ | 10 | 0.8 V | | 41310.5  F cm^−3^ | 165 W cm^−3^ | 3.67  Wh cm^−3^ | This work |
| WMo_2_S_1.5_ | H_2_SO_4_ | 10 | 0.8 V | | 242567  F cm^−3^ | 485.13 W cm^−3^ | 21.56  Wh cm^−3^ | This work |

**Table S3 |** Compositional ratios of synthesised MoS_2_ at different laser fluences

| Element | K-factor | K-factor  type | Absorption correction | wt% | wt%  sigma | Atomic ratio |
| --- | --- | --- | --- | --- | --- | --- |
| N | 3.412 | theory | 1.00 | 0.30 | 0.30 | 0.74 |
| S | 0.975 | theory | 1.00 | 20.19 | 0.48 | 21.84 |
| Mo | 1.763 | theory | 1.00 | 52.57 | 0.76 | 19.00 |
| Total |  |  |  | 100.00 | 0.30 | 100.00 |

**Table S4 |** Compositional ratios of synthesised MoS_2_ at different laser fluences

| Fluence | 1T’/(2H+1T’) | 1T’/(2H+1T’+Mo^5+^) | Mo/S | Mo/O | S/O |
| --- | --- | --- | --- | --- | --- |
| 0.018J/cm^2^ | 0.2658 | 0.23678 | 6.29 | 0.096 | 0.015 |
| .016J/cm^2^ | 0.22233 | 0.19208 | 1.53 | 0.09 | 0.059 |
| 0.014J/cm^2^ | 0.17993 | 0.1757 | 0.97 | 0.061 | 0.063 |
| 0.012J/cm^2^ | 0.1385 | 0.1312 | 0.92 | 0.066 | 0.072 |
| 0.020J/cm^2^ | 0.1526 | 0.14441 | 1.03 | 0.05 | 0.049 |
| 0.024J/cm^2^ | 0.13817 | 0.13179 | 0.93 | 0.061 | 0.051 |

**References**

[1] J. Ye, H. Tan, S. Wu, K. Ni, F. Pan, J. Liu, Z. Tao, Y. Qu, H. Ji, P. Simon, Y. Zhu, *Adv. Mater.* **2018**, *30*, 1.

[2] L. Liu, L. Su, Y. Lu, Q. Zhang, L. Zhang, S. Lei, S. Shi, M. D. Levi, X. Yan, *Adv. Funct. Mater.* **2019**, *29*, 1.

[3] T. Liu, R. Yan, H. Huang, L. Pan, X. Cao, A. DeMello, M. Niederberger, *Adv. Funct. Mater.* **2020**, *30*, 1.

[4] P. Zhuang, Y. Sun, L. Li, M. O. L. Chee, P. Dong, L. Pei, H. Chu, Z. Sun, J. Shen, M. Ye, P. M. Ajayan, *Adv. Mater.* **2020**, *32*, 1.

[5] H. Mahajan, K. U. Mohanan, S. Cho, *Nano Lett.* **2022**, *22*, 8161.

[6] W. Chen, J. Gu, Q. Liu, M. Yang, C. Zhan, X. Zang, T. A. Pham, G. Liu, W. Zhang, D. Zhang, B. Dunn, Y. Morris Wang, *Nat. Nanotechnol.* **2022**, *17*, 153.

[7] K. Prakash, S. Harish, S. Kamalakannan, T. Logu, M. Shimomura, J. Archana, M. Navaneethan, *J. Energy Chem.* **2023**, *80*, 335.

[8] L. Kang, S. Liu, Q. Zhang, J. Zou, J. Ai, D. Qiao, W. Zhong, Y. Liu, S. C. Jun, Y. Yamauchi, J. Zhang, *ACS Nano* **2024**, *18*, 2149.
